# Lack of a Negative Effect of BCG-Vaccination on Child Psychomotor Development: Results from the Danish Calmette Study - A Randomised Clinical Trial

**DOI:** 10.1371/journal.pone.0154541

**Published:** 2016-04-28

**Authors:** Jesper Kjærgaard, Lone Graff Stensballe, Nina Marie Birk, Thomas Nørrelykke Nissen, Kim Thestrup Foss, Lisbeth Marianne Thøstesen, Gitte Thybo Pihl, Andreas Andersen, Poul-Erik Kofoed, Ole Pryds, Gorm Greisen

**Affiliations:** 1 The Department of Paediatrics and Adolescent Medicine, Juliane Marie Centret, Rigshospitalet, Copenhagen University Hospital, Copenhagen, Denmark; 2 The Neonatal Department, Juliane Marie Centret, Rigshospitalet, Copenhagen University Hospital, Copenhagen, Denmark; 3 Department of Paediatrics 460, Copenhagen University Hospital Hvidovre, Hvidovre, Denmark; 4 Department of Neurology, Copenhagen University Hospital Herlev, Herlev, Denmark; 5 Department of Paediatrics, Kolding Hospital, University of Southern Denmark, Kolding, Denmark; 6 Research Center for Vitamins and Vaccines (CVIVA), Bandim Health Project, Statens Serum Institut, Artillerivej 5, Copenhagen, Denmark; Nottingham University, UNITED KINGDOM

## Abstract

**Objectives:**

To assess the non-specific effect of Bacillus Calmette-Guérin (BCG) vaccination at birth on psychomotor development.

**Design:**

This is a pre-specified secondary outcome from a randomised, clinical trial.

**Setting:**

Maternity units and paediatric wards at three university hospitals in Denmark.

**Participants:**

Children born at gestational age (GA) 32 weeks and above. All women planning to give birth at the three sites were invited during the recruitment period. Out of 4262 randomised children, 144 were premature (GA < 37 weeks). There were 2129 children (71 premature) randomised to BCG and 2133 randomised (73 premature) to the control group.

**Interventions:**

BCG vaccination 0.05 ml was given intradermally in the upper left arm at the hospital within seven days of birth. Children in the control group did not receive any intervention. Parents were not blinded to allocation.

**Main outcome measures:**

Psychomotor development measured using Ages and Stages Questionnaire (ASQ) completed by the parents at 12 months. Additionally, parents of premature children (gestational age < 37 weeks) completed an ASQ at 6 and 22 months. Developmental assessment was available for 3453/4262 (81%).

**Results:**

The mean difference in ASQ score at 12 months adjusted for age and prematurity was -0.7 points (BCG vs. control, 95% confidence interval; -3.7 to 2.4), p = 0.67, corresponding to an effect size of Cohen’s *d* = -0.015 (-0.082 to 0.052). The mean difference in ASQ score for premature children at 22 months was -7.8 points (-20.6 to 5.0, p = 0.23), *d* = -0.23 (-0.62 to 0.15).

**Conclusions:**

A negative non-specific effect of BCG vaccination at birth on psychomotor development was excluded in term children.

**Trial Registration:**

ClinicalTrials.gov NCT01694108

## Introduction

The Bacillus Calmette-Guérin vaccine (BCG) against tuberculosis is one of the most commonly used vaccines globally. Approximately 100 million children receive it every year. The vaccine provides some protection against both infection with *M*. *tuberculosis* and progression to tuberculous disease in children[1]. WHO recommends BCG at birth in countries with a high prevalence of tuberculosis[2]. A randomised trial[3] and several observational studies[4–7] have shown that BCG has non-specific beneficial effects resulting in lower mortality and morbidity from infections[8] among BCG vaccinated children in low-income countries. The reduction in mortality and morbidity from infections was larger than what could be explained by prevention of tuberculosis, i.e. a non-specific effect. Also, BCG has been associated with a decrease in atopic disease[9]. Child mortality in Denmark is low but infectious and allergic diseases are common[10]. On that basis, a large randomised trial—The Danish Calmette Study—was conducted to examine if BCG given at birth would reduce childhood morbidity in a high-income country[11].

To our knowledge, the possible non-specific effects of BCG on child psychomotor development have not previously been studied. BCG vaccination induces inflammatory Th1-responses and Th2-responses to unrelated pathogens in vaccinated children[12,13]. BCG also causes localised inflammation at the site of injection for several months[14]. Early life exposure to low-grade inflammation has been linked to development of neuropsychiatric disorders such as cognitive disorders, cerebral palsy, and autism spectrum disorders[15,16]. It has also been associated with increased susceptibility to damage following a hypoxic-ischaemic insult in animal models[17] and with changes in brain maturation in premature sheep[18]. For this reason we wanted to investigate the BCG vaccine’s effect on the neuro-development—particularly relevant for the use of BCG to prevent non-critical illness, and perhaps even more so for premature children who are thought to be more susceptible to inflammatory insults.

Therefore, a secondary outcome of The Danish Calmette Study[11] was to address the question whether BCG at birth could have a negative effect on child development.

## Methods

This study reports on a secondary outcome of The Danish Calmette Study, described in detail elsewhere[11]. Initially we had planned to study the development of premature children only, but as we developed the web-platform for the parental report of child psychomotor development for the premature children at six months, we realised that we would be able to assess the entire cohort using the same approach and we sought and received ethical clearance to do that in time for a 12 months report.

The study was approved by the Committees on Biomedical Research Ethics (J.no. H-3-2010-087), the Danish Data Protection Board (J.no. 2009-41-4141), and the Danish Medicines Agency (J.no. 2612–4356. EudraCT 2010-021979-85. Protocol 2009–323) and registered at www.clinicaltrials.gov (NCT01694108). The trial was supervised by the Good Clinical Practice Units of the Capital Region and the Region of Southern Denmark. The study was monitored by an independent data and safety monitoring board with three members. All parents gave written informed consent.

### Trial methodology in brief

The trial took place at maternity units and paediatric wards at three university hospitals in Denmark. Within seven days of birth, study staff randomised children to receive BCG (Danish SSI strain 1331) in the standard dose of 0.05 mL intra-dermally on the left shoulder or to a no-intervention control group. Parents were not blinded to allocation since the local inflammatory reaction after BCG vaccination could not be mimicked. Randomisation was stratified by prematurity (gestational age [GA] < 37 weeks or ≥ 37 weeks) and children were allocated 1:1 in permuting blocks of 2-4-6 using an online system. Exclusion criteria were GA < 32 weeks, birth weight < 1000 g, child critically ill, known immune-deficiency, no Danish-speaking parent and maternal use of immune modulating drugs during pregnancy. Follow-up consisted of telephone interviews and clinical examination at 3 and 13 months as well as hospital admission diagnoses obtained from national registries at 15 months.

### Follow-up for this study

The Ages and Stages Questionnaire (ASQ) is a series of validated parent-reported, age-related questionnaires ranging from 2 to 60 months, designed to identify children with developmental delay. Each questionnaire contains 30 items on every-day child activities within five sub-domains (communication, fine motor development, gross motor development, problem solving, and personal and social development)[19]. Each item can be checked “yes”, “sometimes”, or “not yet” by the parents. An item is scored 10 for “yes”, 5 for “sometimes”, and 0 for “not yet” and a score for each sub-domain (0 to 60) and a total ASQ score (0 to 300) is calculated if all sub-domains are completed. ASQ is designed to identify children with developmental delay, i.e. those with scores on the left tail of the distribution. Children who score below– 1.5 SD are considered at risk of developmental delay. The distribution of the total ASQ score is truncated to the right when applied to the intended age-group. We wanted to obtain the highest power to detect a difference in mean ASQ score and therefore aimed at a non-truncated distribution of ASQ scores. To obtain this, we used the ASQ intended for children aged 8 months to our premature population at 6 months, the ASQ for 14 months old children to our entire cohort at age 12 months, and the ASQ for 24 months old children for our premature population at 22 months. When using an ASQ for a non-intended age group, it is not possible to determine an optimal ASQ score *a priori*.

ASQ has not been published in Danish. We obtained permission from the publisher to use a version (ASQ 2^nd^ edition) translated for previous research use[20] and adapted it for online use.

We distributed the online ASQ to the parents on the child’s first birthday using an automatically generated email containing a link. We reminded the parents who had not filled in the ASQ at the 13 month telephone and clinical follow-up. Parents of premature children also received an ASQ when the child was 6 and 22 months old. Follow-up took place from April 8^th^ 2013 to August 21^st^ 2015.

### Outcomes

The primary outcome of this study was ASQ score at one year for the entire cohort using complete questionnaires. Sub-group analyses of premature vs. children born to term and boys vs. girls at one year were planned. Secondary outcomes were ASQ score at 6, 12, and 22 months in premature children and ASQ sub-domain scores in the entire cohort at 12 months using complete questionnaires. All analyses were planned prior to un-blinding the data, except an exploratory analysis of the average effect of BCG on premature children at 6, 12, and 22 months which was decided *post hoc*.

### Sample size, data management and statistics

The original sample size of The Danish Calmette Study (4300 children) was determined to be able to detect a 20% reduction in number of hospitalisations up to 15 months with a power of 90%[11]. With this sample size, a significance level of 0.05, and a mean ASQ score of 200 with a standard deviation (SD) of 40, this study has a power of 98% to detect a 5 point difference (effect size 0.13) in mean ASQ score at one year. We expected 5.5% of the cohort to be premature (GA < 37 weeks) yielding a power of 50% to detect a 10 point difference (effect size 0.25) in premature children.

At each follow-up, we grouped parents into *responders* if the questionnaire was complete, *partial responders* if the questionnaire was incomplete and *non-responders* if the questionnaire was missing.

Continuous outcomes were analysed using multiple regression models adjusting for child age at follow-up and for prematurity (binary variable, GA ≥ or < 37 weeks), and raw estimates are presented as well as Cohen’s *d* effect size; calculated as the beta-coefficient divided by the square root of the mean residual variance. The primary analysis was a regression model adjusting for prematurity using complete cases only. Secondary outcomes are reported if all questions within the ASQ sub-domain were answered. A primary outcome sensitivity analyses was conducted adjusting for variables that could be associated with missing outcome; i.e. age at follow-up, child sex, study site, gestational age in days, maternal year of birth, maternal education, multiple birth, maternal smoking during pregnancy, older siblings. Due to a data management system error premature children with gestational age of 32 weeks to 33 weeks and 3 days did not receive the ASQ at 6 months. This mechanism of lost to follow-up was interpreted as missing at random since the probability of being missing was not associated with the outcome. To explore this, we used general/multivariate normal linear models estimated by maximum likelihood to examine the associations between BCG and ASQ score across time for premature children taking into account correlation of measurements within children. The model includes a follow-up variable and an interaction between BCG and follow-up allowing separate effects of BCG to be estimated at 6, 12, and 22 months. Unstructured covariance matrices were used to keep variances and correlations unconstrained. The model is often called Multivariate- or Mixed Model Repeated Measures denoted MMRM.

Slight right-truncation was detected at 12 month follow-up (S1 Fig) but the residuals of the regression were normally distributed, i.e. the assumptions of the primary regression analysis were met. Non-normally distributed discrete data were analysed using Mann-Whitney *U*-test. Categorical outcomes were analysed using χ^2^. The significance level was 0.05 and all tests were two-tailed. All analyses reported are intention-to-treat analyses.

All analyses were performed using STATA 13.1 (StataCorp, Texas, USA). The statistical code used is available in S1 Text.

## Results

**Fig 1:**
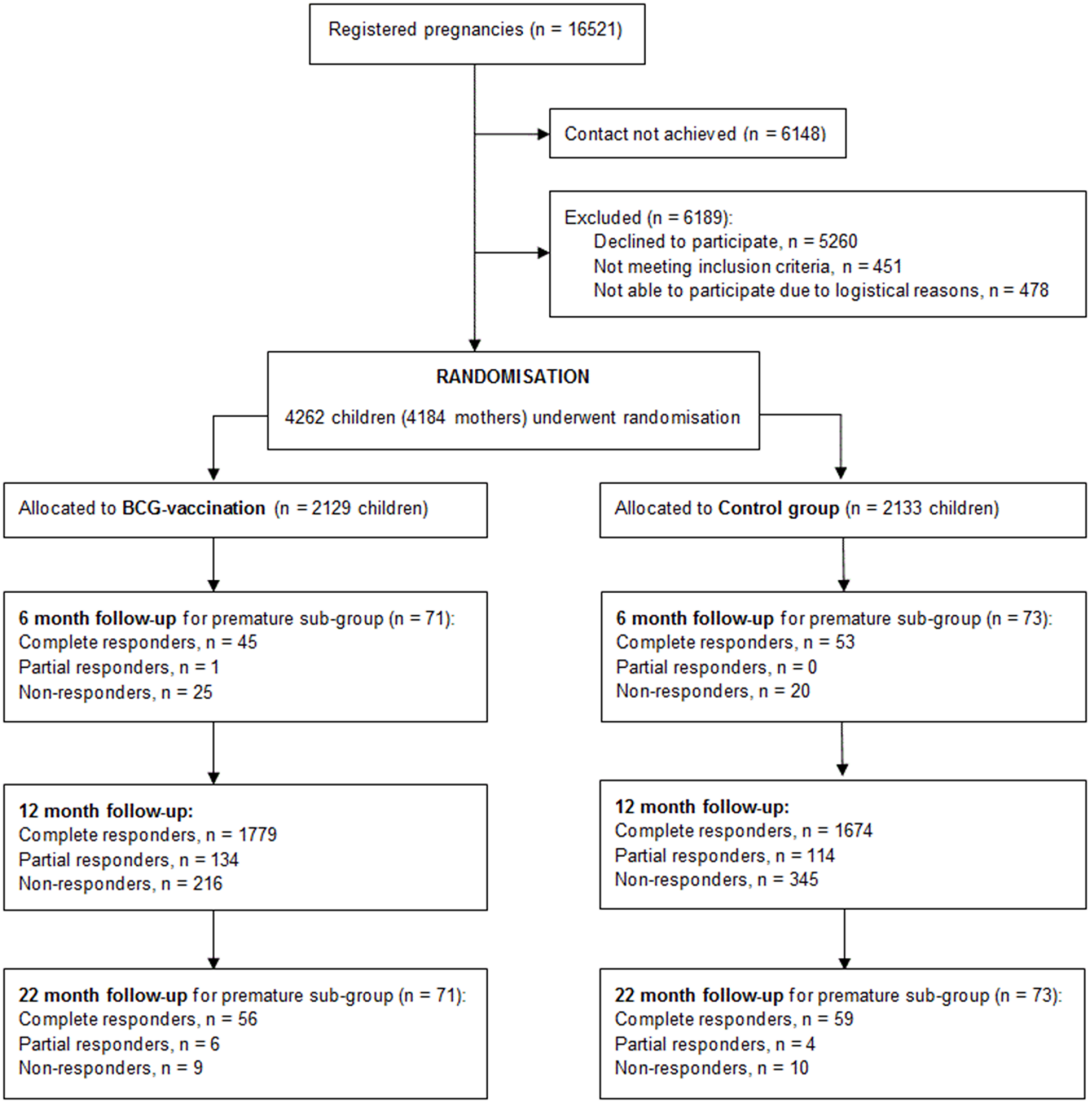
Participant flowchart.

Between October 2012 and November 2013, a total of 16 521 pregnant women were invited. We enrolled 4184 mothers and their 4262 newborn children were randomised (Fig 1). Baseline characteristics were evenly distributed between allocation groups except non-Danish ethnicity and paternal smoking during pregnancy, both of which were more frequent in the control group (Stensballe, 2015). Baseline characteristics grouped by response to follow-up questionnaires are presented in Table 1.

**Table 1:** Baseline characteristics by randomisation group and response to primary outcome follow-up questionnaire at 12 months.

N = 4262		Responders		Partial responders and non-responders	
		(n = 3453)		(n = 809)	
		BCG (n = 1779)	Control (n = 1674)	BCG (n = 350)	Control (n = 459)
**Maternal age, yrs**	Mean (SD)	31.6 (4.5)	31.6 (4.3)	31.1 (5.2)	31.1 (4.7)
**Maternal education**#	N (%)				
Basic schooling and non-theoretical education		367 (20.6%)	328 (19.6%)	98 (28.0%)	114 (24.8%)
Theoretical education incl. BA level		789 (44.4%)	742 (44.3%)	156 (44.6%)	217 (47.3%)
Master level or more		620 (34.9%)	602 (36.0%)	92 (26.3%)	124 (27.0%)
**Maternal smoking during pregnancy** *	N (%)	168 (9.4%)	155 (9.3%)	39 (11.1%)	60 (13.1%)
**Premature, GA < 37 weeks**	N (%)	56 (3.2%)	59 (3.5%)	15 (4.3%)	14 (3.1%)
**Child age at 12 month developmental follow-up, days**	Median	377 (365–447)	378 (365–460)	NA	NA
	(10^th^– 90^th^ percentile)				
**Child sex, boys**	N (%)	922 (52.2%)	888 (53.1%)	176 (50.3%)	249 (54.3%)
**Older biological siblings**	N (%)	727 (40.9%)	664 (39.7%)	178 (50.9%)	192 (41.8%)
**Child exclusively breastfed at three month follow-up**¤	N (%)	1032 (58.0%)	955 (57.5%)	183 (52.3%)	219 (47.7%)
**Multiple birth**	N (%)	53 (3.0%)	62 (3.7%)	15 (4.3%)	22 (4.8%)

^#^ Unknown n = 13,

* Unknown n = 2,

^¤^ Unknown = 41.

SD: Standard deviation, 95% CI: 95% confidence interval, BA: Bachelor, NA: not applicable.

There were 350/2129 (16.4%) partial responders and non-responders in the BCG group vs. 459/2133 (21.5%) in the control group, p < 0.001. Among partial responders and non-responders, there were significantly more women with basic schooling and non-theoretical training (212/809 [26.2%] vs. 695/3453 [20.1%]; p < 0.001), and more families with siblings (370/807 [two were unknown] [45.9%] vs. 1391/3453 [40.3%]; p = 0.004).

There were 46/144 (32.4%) premature partial responders and non-responders at 6 months, 29/144 (20.1%) at 12 months and 22/144 (15.3%) at 22 months.

The mean ASQ score among the 3453 children with complete questionnaires at 12 months was 178.2 points (SD 52.4) in the BCG group vs. 179.9 points (SD 53.6) in the control group. A total of 22 children scored the maximum score of 300.

### Primary and secondary outcomes

The mean difference in ASQ score at 12 months adjusted for age and prematurity was -0.7 points (BCG vs. control, 95% confidence interval [95% CI]; -3.7 to 2.4, p = 0.67), corresponding to an effect size of *d* = -0.015 (-0.082 to 0.052). There were no statistically significant differences in any of the ASQ sub-domains (Table 2). At 22 months, the effect of BCG in premature children was -7.8 points (-20.6 to 5.0), *d* = -0.23 (-0.62 to 0.15). The mean differences in premature children at 6 months and 12 months were also negative but not statistically significant and the effect of BCG was not statistically significantly different between premature and term children at 12 months (Table 3). The results were essentially the same in per protocol analyses (Table 4).

**Table 2:** Primary and secondary outcomes; ASQ score at 12 months.

N = 4262		Mean ASQ score (SD)		Effect of BCG¤ (95% CI)	Cohen’s *d* (95% CI)	P-value
	n	BCG	Controls			
**Primary outcome**						
ASQ sum score	3453	178.2 (52.4)	179.9 (53.6)	-0.7* (-3.7 to 2.4)	**-0.015** (-0.082 to 0.052)	0.67
**Sensitivity analysis**						
ASQ sum score	3453	178.2 (52.4)	179.9 (53.6)	**-0.5**& (-3.4 to 2.5)	**-0.011** (-0.078 to 0.057)	0.76
**Secondary outcomes**						
Communication score	3453	29.2 (13.1)	29.7 (13.3)	-0.3* (-1.0 to 0.5)	**-0.023** (-0.089 to 0.044)	0.51
Fine motor score	3453	37.8 (19.2)	38.0 (19.2)	0.1* (-1.1 to 1.3)	**0.0062** (-0.061 to 0.073)	0.86#
Gross motor score	3453	38.2 (12.6)	38.7 (12.7)	-0.3* (-1.1 to 0.4)	**-0.030** (-0.096 to 0.037)	0.38
Problem solving score	3453	36.6 (13.6)	36.7 (14.1)	0.1* (-0.8 to 1.0)	**0.008** (-0.058 to 0.075)	0.81
Personal and social development score	3453	36.2 (13.3)	36.7 (13.3)	-0.3* (-1.1 to 0.5)	**-0.022** (-0.089 to 0.044)	0.51

* Adjusted for prematurity and age at follow-up,

^&^ Adjusted for prematurity, age at follow-up, child sex, study site, gestational age in days, maternal year of birth, maternal education, multiple birth, maternal smoking during pregnancy, older siblings,

^¤^ Multiple regression beta-coefficients,

^#^ Distribution not normal.

Sensitivity analyses; Mann-Whitney *U* = 0.88, ASQ: Ages and Stages Questionnaire, 95% CI: 95% Confidence interval, BCG: Bacillus Calmette-Guérin vaccine, SD: Standard deviation.

**Table 3:** Subgroup analysis; Total ASQ-score of premature children (gestational age < 37 weeks) at follow-up.

N = 144		Mean ASQ score (SD)		Effect of BCG* (95% CI)	Cohen’s *d* (95% CI)	P-value
Follow-up	n	BCG	Controls			
**6 months**						
Premature children	98	144.8 (48.7)	140.5 (44.1)	**-3.1** (-19.3 to 13.0)	**-0.082** (-0.50 to 0.34)	0.70
**12 months**						
Premature children	115	141.8 (53.0)	153.5 (53.5)	**-11.5** (-28.7 to 5.7)	**-0.26** (-0.64 to 0.13)	0.19¤
Term children	3338	179.3 (51.9)	180.8 (53.3)	**-0.2** (-3.2 to 2.8)	**-0.0044** (-0.072 to 0.064)	0.90¤
**22 months**						
Premature children	122	213.9 (37.4)	224.7 (30.0)	**-7.8** (-20.6 to 5.0)	**-0.23** (-0.62 to 0.15)	0.23

* Multiple regression beta-coefficient adjusted for age at follow-up and gestational age in weeks.

^¤^ P-value for interaction = 0.20,

ASQ: Ages and Stages Questionnaire, 95% CI: 95% Confidence interval, BCG: Bacillus Calmette-Guérin vaccine, SD: Standard deviation.

**Table 4:** Per protocol analyses of primary and secondary outcomes and in a sub-group of premature children.

N = 4262			
Analysis	n	Effect of BCG* (95% CI)	P-value
**Primary**			
ASQ sum score	3423	**-0.6** (-3.6 to 2.5)	0.70
**Secondary**			
Communication score	3650	**-0.4** (-1.1 to 0.4)	0.34
Fine motor score	3644	**0.1** (-1.1 to 1.2)	0.88
Gross motor score	3576	**-0.4** (-1.2 to 0.4)	0.32
Problem solving score	3452	**0.3** (-0.6 to 1.1)	0.56
Personal and social development	3579	**-0.4** (-1.2 to 0.4)	0.37
**Sub-groups:**			
**6 months**			
Premature children	98	**-0.6** (-16.9 to 15.6)	0.94
**12 months**			
Premature children	114	**-11.9** (-28.0 to 4.3)	0.15
Term children	3309	**-0.06** (-3.1 to 3.0)	0.97
**22 months**			
Premature children	121	**-7.9** (-20.8 to 4.9)	0.22

* Multiple regression beta-coefficient adjusted for age at follow-up and gestational age in weeks.

^¤^ P-value for interaction = 0.20,

ASQ: Ages and Stages Questionnaire, 95% CI: 95% Confidence interval, BCG: Bacillus Calmette-Guérin

**Fig 2:**
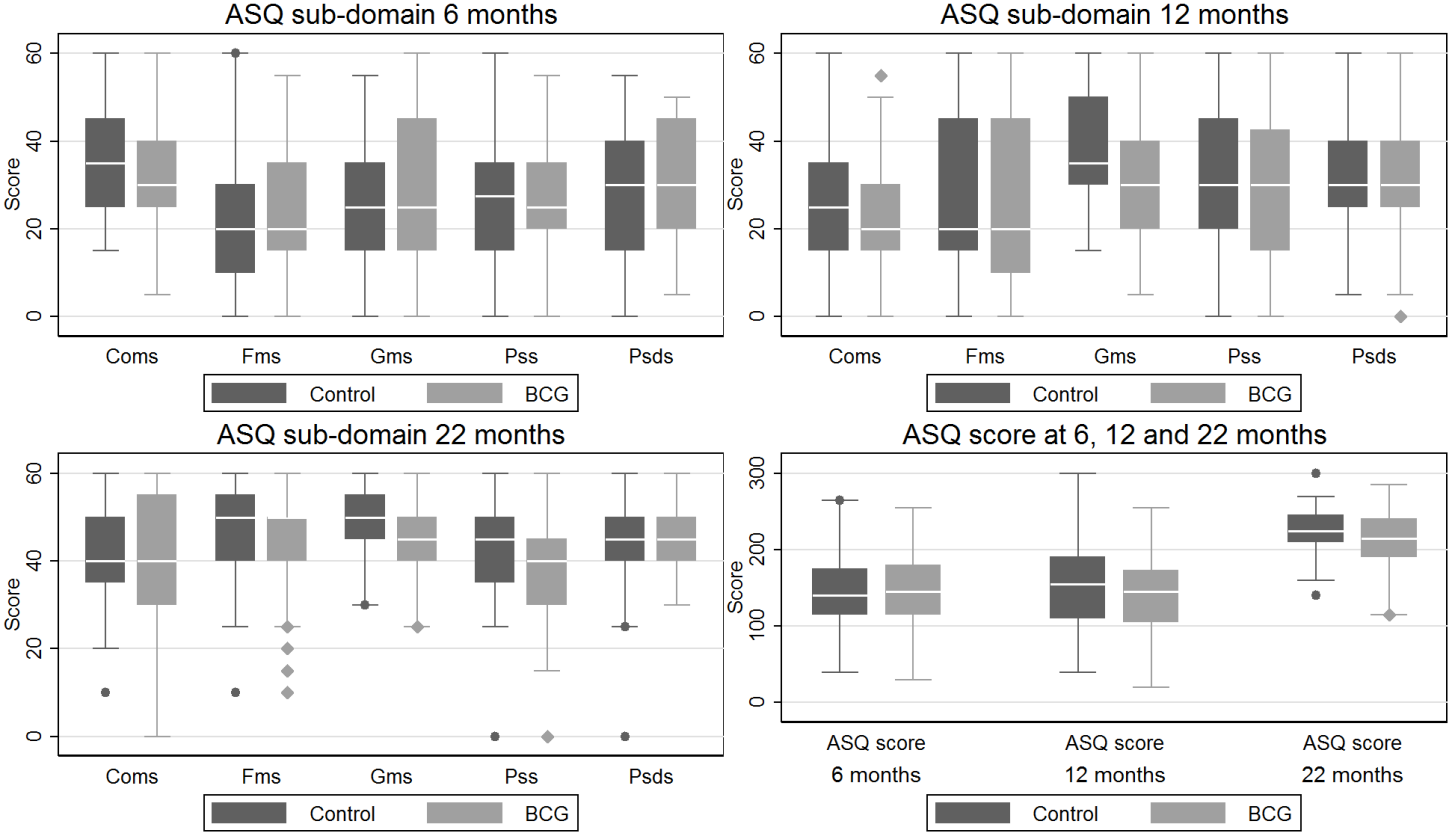
Box-plots of ASQ sub-domain and total scores at 6, 12, 22 months for premature children. ASQ: Ages and stages questionnaire. Coms: Communication score. Fms: Fine motor score. Gms: Gross motor score. Pss: Problem solving score. Psds: Personal and social development score. BCG: Bacillus Calmette-Guèrin vaccine.

In premature children, the effect of BCG did not differ significantly at 6, 12, and 22 months, p = 0.47. The mean effect of BCG was not significant when combining the three estimates; BCG vs. control: -6.4 points (-15.6 to 2.8), p = 0.17. There is no consistent pattern in which sub-domain scores are affected at 6, 12, and 22 months for premature children (Fig 2). Adjustment for factors possibly related to non-response in the sensitivity analysis did not change the estimates significantly (Table 5).

**Table 5:** Sensitivity analyses of ASQ score at 12 months and in the premature subgroup at 6, 12 and 22 months.

		Mean ASQ score (SD)		Mean difference*,BCG vs. control (95% CI)	Cohen’s *d* (95% CI)	P-value
Sensitivity analysis of:	n	BCG	Controls			
**Entire study population**						
At 12 months	3453	178.2 (52.4)	179.9 (53.2)	**-0.5** (-3.4 to 2.5)	**-0.011** (-0.078 to 0.057)	0.76
**Premature children**						
At 6 months	98	144.8 (48.7)	139.0 (45.0)	**-1.9** (-19.8 to 16.1)	**-0.051** (-0.55 to 0.45)	0.84
At 12 months	115	141.8 (53.0)	153.5 (53.5)	**-11.7** (-28.8 to 5.4)	**-0.30** (-0.74 to 0.14)	0.18
At 22 months	122	213.9 (37.4)	224.7 (30.0)	**-4.6** (-18.5 to 9.4)	**-0.14** (-0.58 to 0.29)	0.52

* Regression beta-values adjusted for age at follow-up, child sex, study site, gestational age in days, maternal year of birth, maternal education, multiple birth, maternal smoking during pregnancy, older siblings.

In the entire cohort at one year, the mean, age-adjusted BCG effect for boys was -3.2 points (-7.4 to 0.9), *d* = -0.072 (-0.16 to 0.020) and 2.0 points (-2.4 to 6.4), *d* = 0.045 (-0.052 to 0.14) for girls. The difference in effect between boys and girls was not significant (p = 0.087 for interaction).

## Discussion

In the present study, we report a pre-specified secondary analysis of a randomised, clinical trial, investigating whether BCG vaccination at birth affects the psychomotor development of children. We found no effect of BCG on the psychomotor development at 12 months of age.

The study has sufficient power to detect even a small difference in ASQ score among the mature children and thus has a low risk of not detecting a clinically relevant effect. To measure development, we used a well-known and extensively used questionnaire, the ASQ, which is completed by parents and has been shown to have high validity, sensitivity and specificity as a tool for detecting developmental delay[21]. We succeeded in obtaining a distribution of ASQ-scores with only slight truncation as less than one percent scored the maximum score for the primary outcome and thus obtained the highest possible power to detect a difference in mean score for responders. Also, studying possible non-specific effects using a randomised design yields a low risk of bias. Our study has some limitations that could bias the results towards the null; we had 14.1% non-responders for the primary outcome. Among non-responders, there were more children allocated to the control group, there was a lower level of maternal education, and more families with older siblings. We suspected that non-responders did not return the questionnaires because of one or more of these factors or due to a lower commitment to the study if the child was in the control group[22], i.e. not due to reasons connected with the outcome, but this cannot be ascertained. We believe that the risk of differential drop-out is low since the sensitivity analysis including variables we expected could be associated with drop-out did not change the primary outcome estimate significantly even though the sensitivity analysis cannot control for drop-out associated with the outcome. Another important limitation is the risk of performance bias. Because the parents were not blind to allocation they could underreport adverse events, but we find it implausible that parents would report their child’s development differently based on the allocation in our study, since the parental information sheet (S2 Text) focused on infectious and atopic diseases, i.e. the primary outcomes of The Danish Calmette Study. We only recruited approximately 60% of the intended premature children due to study logistics and increased parental concern regarding participation in research in this group. Finally, it is well-described that the immune response to mycobacterial antigens differs markedly between different strains of BCG[23] and since the effect of BCG on child development is mediated by the immunological response to the vaccine, the findings of this study may not extend to other strains.

Studying potentially negative effects in a trial is only ethically permissible if the expected benefits outweigh the possible side effects. With an expected benefit of a 20% reduction in hospitalisation, we argue that the equipoise required for conducting the trial ethically[24] was present, especially since the existing evidence indicates that premature children benefit the most from the non-specific effects of BCG on mortality[3]. To our knowledge, this is the first study to assess non-specific effects of BCG on psychomotor development. BCG is considered a safe vaccine and the side effects specific to BCG are well described[25–27]. Serious side effects, such as disseminated BCG infection or BCG osteitis, are rare[14] but as knowledge on the immune response generated by BCG as well as on the complex interplay between the immune system and neuro-development increases[28] so does the range of plausible non-specific effects, both beneficial and negative. It has been shown that sensitisation with even a small systemic infectious load can lead to increase susceptibility to brain damage following a hypoxic-ischaemic insult[29] and BCG indeed results in an inflammatory reaction at the injection site which usually lasts for 2–4 months[25,27,30], as a potential source of systemic infection. Two recent reviews concluded that there is a possible link between chronic, low-grade inflammation in early life and the risk of developing an array of neuro-psychiatric disorders in adolescence or adulthood[15,17] and the effect of BCG vaccinating adult patients with early multiple sclerosis only became apparent after 18 months and the effect seemed to increase with time[31]. Thus, our reassuring result at one year of life does not exclude later effects—adverse or beneficial.

In order to corroborate or refute the negative effect estimate we found in premature children, we suggest that neurodevelopmental outcomes are assessed, preferably using ASQ, as part of ongoing and future BCG trials for premature children, even if data would perhaps need to be pooled across studies in order to achieve sufficient power. Perhaps especially so in populations with low prevalence of tuberculosis and a low burden of neonatal infections, i.e. a setting in which the non-specific beneficial effect of BCG on mortality is likely to play a minor role and where prevention of tuberculosis is not a priority at the community level. Conversely, in low-resource settings where a beneficial effect of BCG on all-cause mortality has been demonstrated, the findings from this study should not deter BCG vaccination of premature children, who seem to benefit the most[3].

### Conclusion

A clinically significant negative non-specific effect of BCG vaccination at birth on psychomotor development at 12 months of age was excluded in children born to term. No clear conclusion on effect in premature children (GA < 37 weeks) was possible in the present study.

## Supporting Information

S1 Fig ASQ score by allocation group and age at follow-up. ASQ: Ages and stages questionnaire.
(TIF)

S1 File Study protocol. (DOCX)

S2 File Protocol for the Danish Calmette Study. (PDF)

S3 File Protocol for the Danish Calmette Study (original language). (PDF)

S4 File Consent form for the Danish Calmette Study. (DOCX)

S5 File CONSORT checklist. (DOC)

S6 File TIDieR checklist. (DOCX)

S1 Text STATA code used for statistical analyses in this manuscript. (DOCX)

S2 Text Parental information sheet for the Danish Calmette Study. (DOCX)
