# Supplementary figures and images for: Lack of a Negative Effect of BCG-Vaccination on Child Psychomotor Development: Results from the Danish Calmette Study - A Randomised Clinical Trial
Source: PLoS One. 2016 Apr 28;11(4):e0154541. doi: 10.1371/journal.pone.0154541 (PMC4849633; doi:10.1371/journal.pone.0154541)

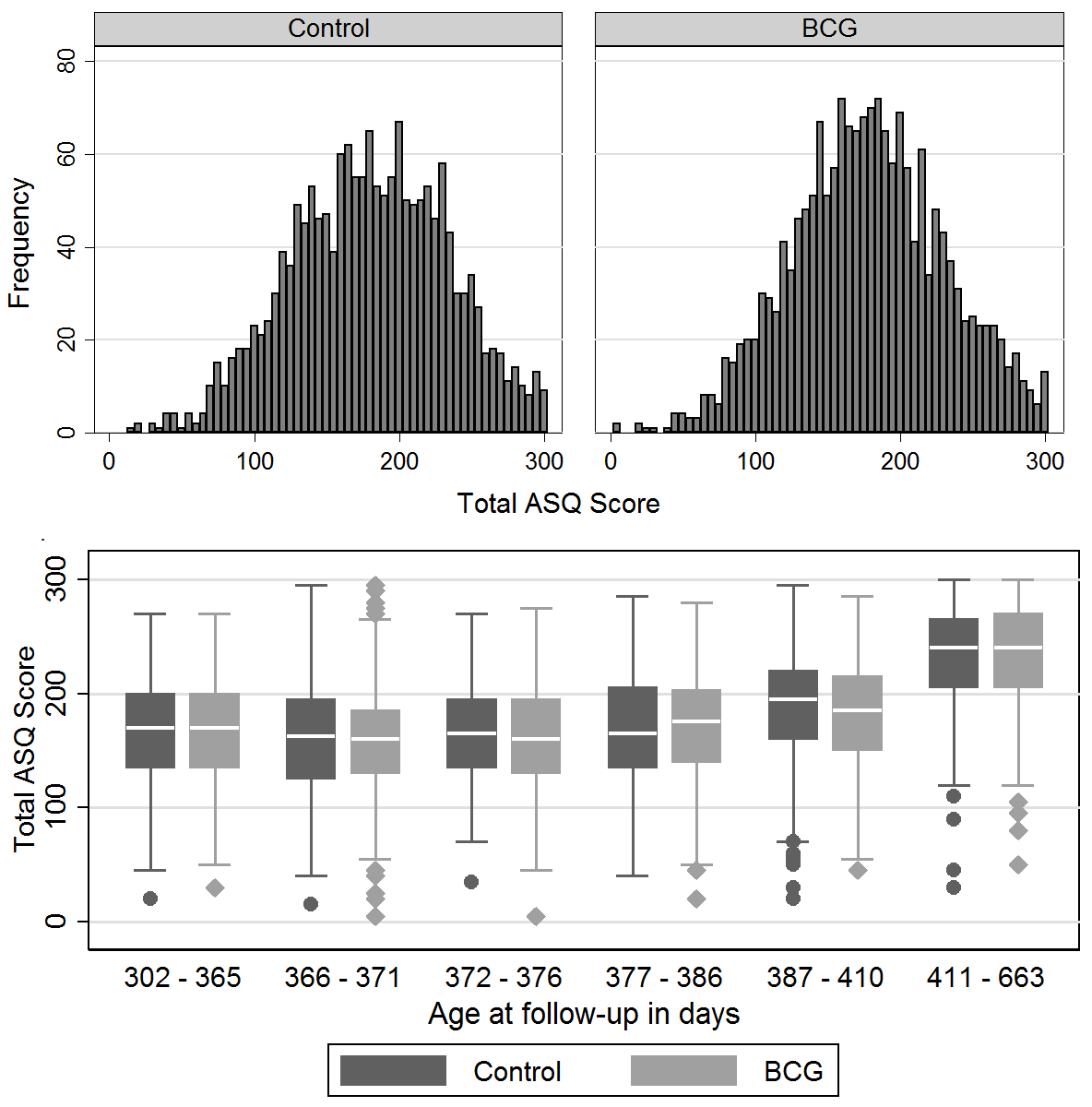

Supplement: S1 Fig — ASQ: Ages and stages questionnaire. (TIF) [file pone.0154541.s001.tif]
