# Supplementary material for: Lack of a Negative Effect of BCG-Vaccination on Child Psychomotor Development: Results from the Danish Calmette Study - A Randomised Clinical Trial
Source: PLoS One. 2016 Apr 28;11(4):e0154541. doi: 10.1371/journal.pone.0154541 (PMC4849633; doi:10.1371/journal.pone.0154541)
Supplement: S1 File — (DOCX) [file pone.0154541.s002.docx]

Sub-study protocol

BCG immunisation at birth and child development

Jesper Kjærgaard, Lone Graff Stensballe, Gorm Greisen

# Summary

Recent studies from Africa have shown that Bacillus Calmette-Guérin (BCG) vaccination is associated not only with specific protection against tuberculosis, but also with so-called non-specific beneficial effects. We recently initiated a large randomised trial of BCG vaccination in Denmark to test the hypothesis that BCG vaccination at birth has beneficial nonspecific effects on child health in Denmark. No less than 4,300 infants born at Rigshospitalet, Hvidovre Hospital and Kolding Sygehus will be randomised to BCG at birth or no vaccine (control group). Follow-up will continue until the children are 13 months of age (22 months for premature infants born before 37 weeks of gestation). Data will be collected using structured telephone interviews and clinical examinations at age 3 and 13 months.

This is a non-inferiority sub-study aiming to examine whether BCG immunisation at birth affects child development.

# Background

As one of the most used vaccines globally, Bacillus Calmette Guérin (BCG) vaccine is administered in > 100 countries to infants at birth to decrease the incidence of severe tuberculosis. Due to the low incidence of tuberculosis in Denmark, the BCG vaccine was withdrawn from the Danish Child Immunisation Programme in 1982. Recently, studies from West Africa have shown positive non-specific effects of early BCG immunisation on childhood morbidity and mortality (1-10). The studies suggest that BCG immunisation protects against various infectious diseases, such as pneumonia, diarrhoea, meningitis and respiratory syncytial virus (7, 11). Danish children below one year of age have high morbidity, primarily from infectious diseases, and resulting increasing use of primary care services and medication (12). Our group therefore initiated a randomised trial of BCG vaccination with the overall aim to test the hypothesis that the BCG-vaccine has positive nonspecific effects in Danish children.

It may be hypothesised that if BCG immunisation at birth results in a lower burden of disease from infections and less use of medicines, BCG immunised children may have better psychomotor development. To our knowledge it has not previously been addressed whether BCG immunisation at birth affects development. In theory, BCG immunisation at birth may also result in a low grade inflammation that may affect the brain negatively, so assessing this outcome will also address an important safety issue. The overall hypothesis of this sub-study is a non-inferiority of BCG-immunised children relating to development. Assessment of development of preterm infants is already part of the Calmette study protocol.

# Research question

Does BCG immunisation at birth affect development in children in the first year of life?

# Methods

## Overall study

## Inclusion

For 1 year all parents planning to give birth at Rigshospitalet, Hvidovre Hospital and Kolding Hospital will receive at letter during 2^nd^/3^rd^ trimester of pregnancy with information on the study and an invitation to participate in the study. The local PhD-students or research midwifes subsequently call the families. They ask the families whether they are willing to participate in the study. A structured interview with the families will be performed. It will be registered on the medical birth records if the family has consented to participation.

### Exclusion

Infants born before gestational age 32 weeks and/or birth weight < 1000g, infants with known congenital disease, anomaly or malformation, immune deficiency and HIV, will be excluded. Non-Danish speaking parents will be excluded.

*Intervention*

Children born to mothers, who have accepted to participate, will be randomised to either intervention group or to the control group at birth. Block-randomisation stratified by gestational age (≥ 37 weeks of gestation vs. < 37 weeks of gestation) will be performed electronically just before vaccination by the overall study electronic case report system (e-crf).

Vaccination

Children randomised to the BCG vaccination group will receive an intradermal BCG vaccine (Statens Serum Institute “BCG vaccine”) in the standard dose 0.05 ml in the upper, lateral part of the shoulder of the child by specially trained midwives.

### Data collection

At the inclusion and at 3 and 13 months after birth structured telephone interviews will be conducted. These will focus on the infants’ anthropometric measurements, nutrition, visits at the general practitioner, hospitalisations and infectious illnesses. The parents will be invited for clinical examinations of the child at ages 3 and 13 months.

Figure 1 outlines the study design (in Danish).


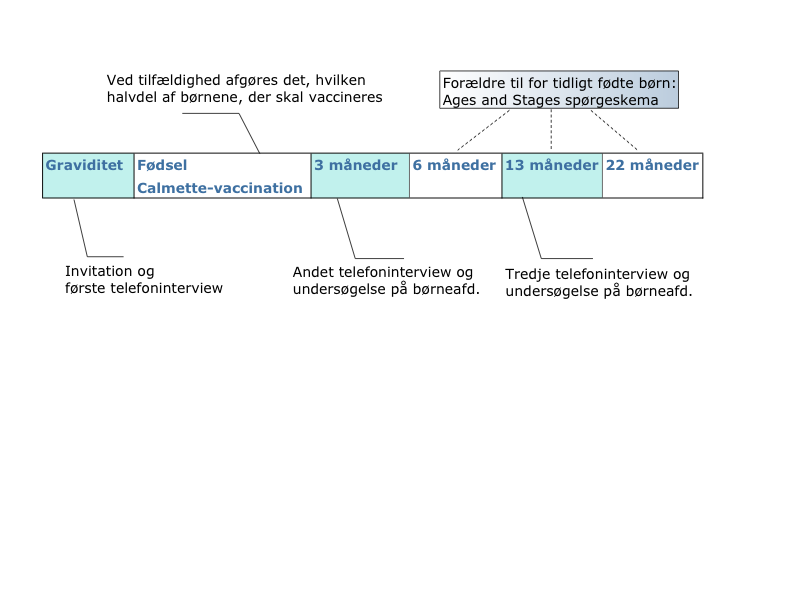


**Emailinvitation til at deltage i børns udvikling sub-studie**

Figure 1: Study design

## Sub-study outcome

Ages and Stages Questionnaire (agesandstages.com): A validated, parent reported questionnaire concerning development in five domains: Communication, gross motor, fine motor, problem solving and social development. The questionnaire results in a combined score (0 - 300) that assesses the overall psychomotor development of the child. The questionnaire will be distributed to all parents of 13 months old children born at term (Appendix 1). Parents will automatically receive an email asking whether they would like to participate in this sub-study when the child is approximately 12.5 months old. The email will contain a link that directs to a web based version of ASQ and parents type the information directly into the electronic case report form.

# Sample size considerations

The overall trial

The main trial includes 4300 children to be able to detect a 20% reduction in infant hospitalisation with 90% power and α = 0.05.

Sub-study

The entire Calmette-cohort will comprise 4300 children. Of these approximately 300 will be preterm (< 37 GA). Based on a sample size of 2000 mature children in each group, an α = 0.05, a mean ASQ score of 200 with a standard deviation (SD) of 40 this study will have a power of approximately 97% to detect a 5 points difference (effect size 0.12) between mean scores in BCG vaccinated children compared to unvaccinated children. When used for screening purposes, an ASQ score of ≥1.5 SD (approximately 60 points at age 13 months) below the mean is considered indication of developmental delay (13). The high power of this sub-study will allow meaningful exploratory subgroup analyses.

## Adjustment

As the randomisation is stratified for gestational age all the statistical analyses will adjust for this factor. All main results will be presented overall and separately for girls and boys since a strong sex-differential effect of vaccines on mortality and hospitalisations has previously been found

# Statistics

**Data source:** Ages and Stages Questionnaire

**Primary outcome:** Age-adjusted sum score of ASQ at 13 months.

**Secondary outcomes:** Sub-scale scores (communication, fine motor, gross motor, problem solving, personal and social development).

**Sensitivity analyses:**

We expect to have data on approx. 85% of enrolled children. Some of these data may be incomplete. The ASQ manual suggests that partially completed scores can be used to impute the total score. The equation is: total ASQ score = sum of ASQ sub-scores / (300 - 10 * no. of missing)*300. In case of questionnaires less than 66% complete, they will count as missing. Sensitivity analyses using the simple imputation-data will be conducted.

Completely missing scores will be imputed using multiple imputations. Questionnaires less than 66% filled will be set to missing and then imputed.

Excluding children that did not follow the allocation.

**Exploratory outcomes:** Correlation of age-adjusted ASQ sum-score with sum of parent reported illness. Exploratory analyses will be presented with crude p-values not corrected for multiple comparisons.

**Statistical analysis:**

1. Regression analysis of ASQ scores with BCG, prematurity and age at follow-up as independent variables.
2. Secondary outcomes subject to post-hoc Holland correction.
3. If data are not reasonably normally distributed, we will present confidence intervals obtained by bootstrapping.

**Subgroup analyses:** Premature children, sex, BCG-immunisation within two days of birth. Subgroup analyses will be conducted in case of both statistically significant and statistically insignificant primary outcome.

**Potential effect modifiers that can be considered in subgroup analyses:**

- Maternal age
- Maternal education
- Ethnicity (Danish/non-Danish)
- Parental smoking (yes/no)
- BCG with scar, BCG without scar, and control group.
- Parental atopy (eczema, asthma, allergic rhinoconjunctivitis)
- Maternal BCG (yes/no)
- Smoking during pregnancy (yes/no)
- Mode of delivery (Vaginal/Caesarean section)
- Antibiotics given to the mother during labour (yes/no)
- Birth weight (continuous)
- Breastfeeding (exclusively breast fed at three months of age (yes/no)).
- Vaccination history (vaccination before examination at 3 and 13 months)
- Batch no. (1/2)
- Infanrix Hexa (yes/no)
- Pets (yes/no)
- Siblings (yes/no)
- Day care (yes/no)

These other modifiers will possibly be explored. Data will be available on request. If very influential modifiers are found in the main study, these will be examined post-hoc.

# Ethics

The main trial has been approved by the Danish Data Protection Board (J.no. 2009-41-4141), the Committees on Biomedical Research Ethics (J.no. H-3-2010-087), and the Danish Medicines Agency (J.no. 2612-4356. EudraCT 2010-021979-85. Protocol 2009-323). The trial is supervised by the Good Clinical Practice (GCP) Units in the Capital Region of Denmark and the Region of Southern Denmark. The study is monitored by the GCP.

# Study frames of the overall study

The study will be performed in a collaboration between Rigshospitalet, Hvidovre Hospital, Kolding Sygehus Lillebælt and Research Centre for Vitamins and Vaccines (CVIVA) which is funded by Danish National Research Foundation.

At Rigshospitalet 5,000 give birth per year, at Hvidovre Hospital 6,000 and Kolding Hospital 4,000 Even if only 1/3 of the expected births per year are included during the inclusion period of one year, the study will be powered according to the study plans.

MD PhD Lone Graff Stensballe, Rigshospitalet is study sponsor and supervises and arranges the collaboration between the 3 departments of inclusion. Prof. Gorm Greisen and Lone Graff Stensballe will supervise the study at Rigshospitalet, Prof. Ole Pryds will supervise at Hvidovre Hospital, and professor Poul-Erik Kofoed will supervise at Kolding. Each place of inclusion will collect and share data.

# Study group

## Supervisors of the present study

Prof. Dr.Med.Sci, Gorm Greisen, Neonatal Clinic, Rigshspitalet and MD, PhD, Lone Graff Stensballe, BørneUngeKlinikken, Rigshospitalet.

# The study will be carried out by

MD, PhD-student, Jesper Kjærgaard, BørneUngeKlinikken, Rigshospitalet.

# Time line

The overall study started October 2012. This sub-study will be conducted within the frames of Jesper Kjærgaards present PhD study. The first child enrolled will be 13 months in November 2013. Data collection will then continue for approximately 15 months, leaving 8 months for data management, analyses and paper writing.

# Publication

The study will be published in a peer-reviewed international Journal with Jesper Kjærgaard as first author, Lone Graff Stensballe as second author and Gorm Greisen as last author.

# Reference List

1. Aaby P, Jensen H, Garly ML, Bale C, Martins C, Lisse I. Routine vaccinations and child survival in a war situation with high mortality: effect of gender. Vaccine. 2002 Nov 22;21(1-2):15-20.

2. Aaby P, Jensen H, Gomes J, Fernandes M, Lisse IM. The introduction of diphtheria-tetanus-pertussis vaccine and child mortality in rural Guinea-Bissau: an observational study. Int J Epidemiol. 2004 Apr;33(2):374-80.

3. Aaby P, Shaheen SO, Heyes CB, Goudiaby A, Hall AJ, Shiell AW, et al. Early BCG vaccination and reduction in atopy in Guinea-Bissau. Clin Exp Allergy. 2000 May;30(5):644-50.

4. Aaby P, Vessari H, Nielsen J, Maleta K, Benn CS, Jensen H, et al. Sex differential effects of routine immunizations and childhood survival in rural Malawi. Pediatr Infect Dis J. 2006 Aug;25(8):721-7.

5. Garly ML, Martins CL, Bale C, Balde MA, Hedegaard KL, Gustafson P, et al. BCG scar and positive tuberculin reaction associated with reduced child mortality in West Africa. A non-specific beneficial effect of BCG? Vaccine. 2003 Jun 20;21(21-22):2782-90.

6. Kristensen I, Aaby P, Jensen H. Routine vaccinations and child survival: follow up study in Guinea-Bissau, West Africa. BMJ. 2000 Dec 9;321(7274):1435-8.

7. Roth A, Gustafson P, Nhaga A, Djana Q, Poulsen A, Garly ML, et al. BCG vaccination scar associated with better childhood survival in Guinea-Bissau. Int J Epidemiol. 2005 Jun;34(3):540-7.

8. Roth A, Jensen H, Garly ML, Djana Q, Martins CL, Sodemann M, et al. Low birth weight infants and Calmette-Guerin bacillus vaccination at birth: community study from Guinea-Bissau. Pediatr Infect Dis J. 2004 Jun;23(6):544-50.

9. Roth A, Sodemann M, Jensen H, Poulsen A, Gustafson P, Weise C, et al. Tuberculin reaction, BCG scar, and lower female mortality. Epidemiology. 2006 Sep;17(5):562-8.

10. Velema JP, Alihonou EM, Gandaho T, Hounye FH. Childhood mortality among users and non-users of primary health care in a rural west African community. Int J Epidemiol. 1991 Jun;20(2):474-9.

11. Stensballe LG, Nante E, Jensen IP, Kofoed PE, Poulsen A, Jensen H, et al. Acute lower respiratory tract infections and respiratory syncytial virus in infants in Guinea-Bissau: A beneficial effect of BCG vaccination for girls: Community based case-control study. Vaccine. 2005 Jan 26;23(10):1251-7.

12. Johansen A, Jespersen LN, Davidsen M, Michelsen SI, Morgen CS, Helweg-Larsen K, et al. Danske børns sundhed og sygelighed. Copenhagen: Statens Institut for Folkesundhed; 2009 February 2009.

13.  Bricker D, Squires J. Ages & Stages Questionnaires: A Parent-Completed, Child-Monitoring System. 2nd ed. Paul H. Brookes Publishing Co; Baltimore, MD: 1999.
