# Supplementary material for: Lack of a Negative Effect of BCG-Vaccination on Child Psychomotor Development: Results from the Danish Calmette Study - A Randomised Clinical Trial
Source: PLoS One. 2016 Apr 28;11(4):e0154541. doi: 10.1371/journal.pone.0154541 (PMC4849633; doi:10.1371/journal.pone.0154541)

BCG vaccination og sygelighed blandt danske småbørn

En prospektiv, randomiseret, klinisk lægemiddelafprøvning

Vi bekræfter hermed med vores underskrift, at denne version af studie-protokollen er den gældende, og at vi vil udføre studiet i henhold hertil.

Dato Underskrift

Lone Graff Stensballe, sponsor og investigator på Rigshospitalet

Dato Underskrift

Ole Pryds, investigator på Hvidovre Hospital

Dato Underskrift

Poul-Erik Kofoed, investigator på Kolding Sygehus

Introduktion

Bacille Calmette-Guérin (BCG) vaccinen er en af de mest anvendte vacciner i verden. BCG vaccinen gives i øjeblikket i mere end 100 lande verden over til spædbørn ved eller umiddelbart efter fødslen med det formål at forebygge alvorlig infektion med tuberkulose (WHO. *Issues relating to the use of BCG in immunization programmes*. A discussion document (unpublished document WHO/V&B/99.23; available from Vaccines and Biologicals, World Health Organization, 1211 Geneva 27, Switzerland). Vaccinen giver beskyttelse mod de alvorligste former for tuberkulose, som især er meningitis hos helt små børn.

I Danmark ophørte man at bruge BCG-vaccination i børnevaccinationsprogrammet i begyndelsen af 1980'erne, da forekomsten af tuberkulose i Danmark var lav. Tuberkulose er imidlertid tiltagende udbredt globalt og bl.a. i Grønland BCG vaccineres alle nyfødte.

Studier fra Vestafrika viser at BCG vaccination også har en ikke-specifik effekt, der nedsætter dødelighed og sygelighed blandt små børn i de første leveår (1-10). I to randomiserede studie blandt børn med lav fødselsvægt, som normalt ikke får BCG ved fødslen, var neonatal dødelighed 48% lavere i gruppen som fik tidlig BCG ved fødslen sammenlignet med de børn der fik BCG senere som det har været normal praksis (11;12). Den positive effekt var særlig udtalt blandt de mest skrøbelige børn med fødselsvægt på mindre 1500 g (11).

Børn under et år har høj sygelighed og som følge heraf mange lægebesøg, et højt medicinforbrug og mange indlæggelser (http://www.si-folkesundhed.dk/upload/web_susy_b%C3%B8rn.pdf). Gennem tre årtier har forekomsten af de atopiske sygdomme, astma, allergi og eksem, desuden været støt stigende i høj-indkomstlande verden over. Således er astma og eksem nu de hyppigst forekommende kroniske sygdomme blandt børn i høj-indkomstlande, og 25% af den voksne befolkning lider af astma, høfeber eller allergi. Mange studier har forsøgt at afdække årsagerne hertil. Flere har peget på at immunologisk under/fejl-stimulering som følge af en tilværelse i tiltagende hygiejniske omgivelser med færre infektioner, den såkaldte hygiejne-hypotese, kan være en del af forklaringen (13). En velkendt, ikke specifik effekt af BCG vaccinen er at medføre en stærk immunologisk påvirkning af immunforsvaret, som bl.a. medfører et kraftigt interferon gamma (IFN-γ) respons (14). Dette respons vides at balancere det såkaldte T-hjælper celle 2 (TH2) respons, der ses blandt individer med tendens til de såkaldte atopiske sygdomme (15). På den baggrund er der gennem de seneste 10 år lavet en række mindre studier, både i høj- og lav-indkomstlande, hvor man har forsøgt at undersøge om individer, der er blevet BCG vaccinerede, har nedsat forekomst af

de atopiske sygdomme. Resultaterne har været divergerende, idet nogen ikke har kunnet påvise nogen beskyttende effekt af BCG vaccinen, mens andre har peget på en beskyttende effekt (16-33). Studierne har retrospektive, ikke-randomiserede studier. Så vidt vi ved, er der kun et studie, der hidtil har undersøgt BCG-vaccinens effekt i et høj-indkomstland i et randomiseret design. Det var et mindre hollandsk studie, der fandt, at BCG vaccine reducerede forekomsten af atopisk dermatitis med omkring 40% (34).

Der mangler nu at blive lavet et stort prospektivt kohortestudie med styrke nok til endeligt at undersøge en mulig positiv ikke-specifik effekt af BCG-vaccination mod sygelighed, herunder atopisk sygdom blandt små børn i høj-indkomstlande.

Formål

Formålet er at undersøge om danske spædbørn, der bliver BCG-vaccinerede lige efter fødslen, har en reduceret sygelighed, herunder færre hospitalsindlæggelser, mindre medicin-forbrug og en reduceret risiko for at udvikle de udbredte atopiske sygdomme, astma og eksem.

Hovedformål

At undersøge om BCG-vaccinerede spædbørn har færre hospitalsindlæggelser i første leveår.

Sekundære formål

1. At undersøge om BCG vaccinerede spædbørn bruger mindre antibiotika i første leveår.
2. At undersøge om BCG vaccinerede spædbørn udvikler mindre atopisk sygdom (eksem, hvæsen, astma og allergi) og bruger mindre medicin mod atopisk sygdom i første leveår.
3. At undersøge ovennævnte specifikt blandt gruppen af præmature børn født med gestationsalder uge 32+0 til 36+6 og blandt gruppen af børn med lav fødselsvægt ≥1000g til <2500g.
4. At undersøge, hvorvidt BCG vaccination ved fødslen modulerer det immunologiske system målt som antal hvide blodlegemer, herunder T-celle sub-populationer og profiler, cytokin-produktion og thymusstørrelse ved 3 og 13 måneders alderen, og tillige ved 13 måneder målt som immunglobuliner og antistof-respons på vaccinerne, som gives via Børnevaccinationsprogrammet.

Patienter og metoder

Inklusion

I en periode på cirka 1 ½ år fra september 2012 til 2014 vil forældre til alle nyfødte på tre af landets store fødeafdelinger, Rigshospitalet (antal fødsler årligt 4000 til 5000), Hvidovre Hospital (antal fødsler årligt knap 6000) og Kolding Sygehus (antal fødsler årligt knap 4000), blive tilbudt at deres nyfødte barn randomiseres til BCG givet i ugen efter fødslen eller ingen vaccine. Studiet stiler mod at inkludere i alt 4300 spædbørn. Børnene vil blive fulgt i registre til de er 15 måneder, forældre til præmature børn vil dog få et brev med et afsluttende spørgeskema når de præmature børn er knap to år. Se venligst Figur 1 som illustrerer studiets tidsforløb.

Inklusionskriterier

- Gestationsalder ≥ 32 uger og fødselsvægt ≥ 1000 g.
- Barnet skønnes at være velskabt og fuldt levedygtigt.
- Barnet har ingen tegn på medfødt immundefekt.
- Skriftligt samtykke fra begge forældre (dog kun moderen i fald der ikke er kendt far) foreligger.

Eksklusion

Alle børn født før 32. gestationsuge og/eller med FV < 1000gram, og børn med kendt immundefekt herunder HIV og kromosomdefekt, ekskluderes. Syge børn med behov for intensiv behandling ekskluderes.

Randomisering.

Studiets dataindsamling foregår i et elektronisk case-report-form-system (e-crf) i henhold til international standard. Når barnet er født, indtastes barnets CPR i dette system, og barnet blok-randomiseres elektronisk til BCG-vaccine eller ingen behandling. Blokrandomiseringen er stratificeret for gestationsalder (≥ 37 ugers gestationsalder vs. < 37 ugers gestationsalder (præmatur)) og for hospital (Rigshospitalet/Hvidovre Hospital/Kolding Sygehus).

Eksposure

Intradermal BCG vaccination (0.05 ml af vaccine fra Statens Serum Institut (SSI), Danmark) i overarmen svarende til insertionen af musculus deltoideus. Vaccinationen udføres af en trænet person og eventuelt ubehag imødegås ved peroralt sukkervand før proceduren.

Produktresumé for BCG-vaccinen

Lægemiddelstyrelsens produktresumé for BCG vaccinen fra SSI findes på http://www.produktresume.dk/docushare/dsweb/GetRendition/Document-13163/html

Sikkerhed ved håndtering af BCG-vaccinen (forsøgsbehandlingen)

BCG-vaccinerne der skal anvendes vil blive håndterede i fuld overensstemmelse med forskrifterne i produktresumeet, herunder

- Må ikke blandes med andre lægemidler.
- Opbevaret i max 18 mdr. ved 2-8 grader i original emballage beskyttet mod lys.
- Anvendes max 4 timer efter re-konstitution.

Baggrundsoplysninger

Ved inklusion indsamles via et struktureret interview med forældrene oplysninger om fødslen, allergiske sygdomme i familien, rygning og kæledyr (Vedlagt som bilag). I Kolding vil alle endvidere blive stillet spørgsmål om det at lade sit barn vaccinere (Vedlagt som bilag).

Udfald (kildedata)

Primært outcome er spædbarnets morbiditet målt som antal hospitalsindlæggelser.

Antal hospitalskontakter og lægemiddelforbrug registreret i de danske registre

Opfølgning i registre

De inkluderede børn vil blive fulgt i Landspatientregisteret for antal indlæggelser (primært outcome) og ambulante kontakter med sygehusvæsenet.

De inkluderede børn vil blive fulgt i Lægemiddelregisteret for samlet forbrug af medicin, herunder forbrug af antibiotika, anti-astmatisk medicin, og hudmidler mod eksem.

Denne registeropfølgning vil vare til børnene er 15 måneder.

Ved at opsøge outcome i registre sikres at outcome registreres af personer udenfor studiegruppen, helt uafhængigt af eksposure, hvilket minimerer risikoen for bias.

Landspatientregisteret

Landspatientregisteret (LPR) har siden 1977 registreret oplysninger om alle kontakter med sygehusvæsenet i den danske befolkning (35). Fødselsregisteret har siden 1995 hørt under LPR. Siden 1994 har LPR benyttet diagnosekoder fra "International Statistical Classification of Diseases and Related Health Problems" (ICD10). I ICD10-systemet findes specifikke diagnosekoder for hoved og bi-diagnoser for hver enkelt kontakt en person har haft med sygehusvæsenet.

For studiepopulationen, dvs. BCG-vaccinerede børn, fra kontrol-børn og desuden som en ekstra kontrolgruppe alle øvrige danske børn, der fødes i studieperioden, indhentes fra LPR oplysning om samtlige kontakter med sygehusvæsenet. Den ekstra kontrolgruppe følges alene via register-oplysninger, og kontaktes ikke i forbindelse med studiet.

Som primært mål for sygelighed opgøres hospitaliseringsraten for BCG-vaccinerede børn og kontrol-børn og sammenlignes i overlevelsesanalyse. Desuden sammenlignes hospitaliseringsraten for forskellige specifikke sygdomme, herunder infektionssygdomme og de atopiske sygdomme.

Fra Fødselsregisteret indhentes oplysninger om mulige confoundere/effekt modifikatorer: Gestationsalder, fødselsvægt, fødselstype, maternel rygning og om forældrene er samboende ved fødslen.

Lægemiddelregisteret

(http://www.dkma.dk/1024/visUKLSArtikel.asp?artikelID=743)

Lægemiddelregisteret indeholder CPR-nummerbaserede oplysninger om salg af samtlige receptpligtige medikamenter i Danmark registreret ifølge ATC-kode-systemet (Anatomical Therapeutical Chemical Classification System). Herfra indhentes information om alle børnenes forbrug af medicin.

Som primært mål for medicinforbrug opgøres kumulerede antal indløste recepter for BCG-vaccinerede børn og kontrol-børn og sammenlignes. Desuden sammenlignes antal indløste recepter for medicin mod forskellige specifikke sygdomme, herunder infektionssygdomme og de atopiske sygdomme.

Håndtering og arkivering af register-kildedata

Rå-kopi af data fra ovennævnte registre opbevares sikret i henhold til Datatilsynets forskrifter og når data er linket via CPR-numre bliver der arbejdes elektronisk i henhold til sikkerhedsforskrifter med en anonymiseret kopi af data.

Morbiditeten blandt børnene måles desuden som nævnt nedenfor (se også Figur 3):

Interview 1

Ved 3 måneders alderen, før barnet får flere vacciner ifølge det danske vaccinationsprogram, laves et struktureret telefoninterview med en af forældrene. Ved interviewet indhentes oplysninger om antropometri, antal sundhedsplejerske-besøg, besøg hos egen læge og indlæggelser (Interviewet er vedlagt som bilag). Ved interviewet adspørges forældrene om de vil møde op til en kort klinisk undersøgelse af deres barn (se nedenfor, undersøgelses-skema vedlagt).

Klinisk og paraklinisk opfølgning 1

Alle forældre vil blive tilbudt en klinisk undersøgelse af deres barn, når barnet er 3 måneder gammelt og før barnet er vaccineret ifølge det danske vaccinationsprogram. Ved undersøgelsen vil barnet blive målt og vejet, lyttet på hjerte og lunger, og lægen vil se efter tegn til hvæsen og eksem. På Hvidovre Hospital vil man endvidere spørge forældrene om lov til at tage en blodprøve på ti milliliter fuldblod til med henblik på undersøgelse af den immunologiske effekt af vaccinen. Når der er indsamlet 150 blodprøver blandt BCG vaccinerede børn, og 150 blodprøver blandt ikke-BCG-vaccinerede børn, vil indsamlingen af blodprøver ophøre (se venligst afsnit om studie-størrelse nedenfor). Blodprøverne vil blive analyseret for leukocytter, herunder T-celle sub-populationer og profiler og cytokin-produktion. Resten af prøverne vil herefter blive sikkert opbevaret i fryseskab i 5 år efter projektets afslutning i biobank med henblik på mulige fremtidige analyser i danske laboratorier, da det er sandsynligt at der i løbet af de næste år udvikles nye metoder at måle immunforsvarets funktion på.
På Hvidovre Hospital vil man også spørge forældrene om lov til at ultralydsscanne børnenes thymus (brissel) (36). Når i alt 300 børn har fået ultralydsscannet deres thymus (150 BCG vaccinerede og 150 ikke-BCG-vaccinerede) vil man ikke behøve flere børn til thymus-scanninger og denne undersøgelse vil ophøre. Det vil tilstræbes at det er de samme børn, som deltager i blodprøvetagning og ultralydsscanning.

Interview 2

Igen ved 13 måneders alderen laves struktureret telefoninterview med en af forældrene. Ved interviewet indhentes oplysninger om antropometri, antal sundhedsplejerske-besøg, besøg hos egen læge, indlæggelser, og barnets vaccinationsdækning ifølge Det Danske Børnevaccinationsprogram (Interviewet er vedlagt som bilag). Ved interviewet adspørges forældrene om de vil møde op til en kort klinisk undersøgelse af deres barn (se nedenfor, undersøgelses-skema vedlagt).

Klinisk og paraklinisk opfølgning 2

Alle forældre vil blive tilbudt en klinisk undersøgelse af deres barn, når barnet er 13 måneder gammelt. Ved undersøgelsen vil barnet blive målt og vejet, lyttet på hjerte og lunger, og lægen vil se efter tegn til hvæsen og eksem.
På Hvidovre Hospital vil man endvidere igen spørge forældrene om lov til at tage en blodprøve på ti milliliter fuldblod til med henblik på undersøgelse af den mulige immunologiske effekt af BCG-vaccinen. Når der er indsamlet 150 blodprøver blandt BCG vaccinerede børn, og 150 blodprøver blandt ikke-BCG-vaccinerede børn, vil indsamlingen af blodprøver ophøre (se venligst afsnit om studie-størrelse nedenfor). Blodprøverne vil blive analyseret for hvide blodlegemer, herunder T-celle sub-populationer og profiler, cytokin-produktion, immunglobuliner og specifikke antistoffer mod vaccinerne givet ifølge Det Danske Børnevaccinationsprogram (37). Resten af prøverne vil herefter blive sikkert opbevaret i fryseskab i 5 år efter projektets afslutning i biobank med henblik på mulige fremtidige analyser i danske laboratorier, da det er sandsynligt at der i løbet af de næste år udvikles nye metoder at måle immunforsvarets funktion på.
På Hvidovre Hospital vil man også spørge forældrene om lov til at ultralydsscanne børnenes thymus (brissel) (36). Når i alt 300 børn har fået ultralydsscannet deres thymus (150 BCG vaccinerede og 150 ikke-BCG-vaccinerede) vil man ikke behøve flere børn til thymus-scanninger og denne undersøgelse vil ophøre. Det vil tilstræbes at det er de samme børn, som deltager i blodprøvetagning og ultralydsscanning.

Interview 3 (ASQ, Ages & Stages Questionnaire - Kun til præmature børn)

For præmature børn født med gestationsalder uge 32+0 til 36+6 vil der når børnene er 6, 13 og 22 måneder gamle per email blive tilsendt forældrene et link til ASQ-spørgeskema, som en af

forældrene vil blive bedt om at udfylde. Svarene fra dette skema vil gøre det muligt at belyse om BCG vaccinen skulle have særlig betydning for de potentielt mere sarte præmature børns udvikling (Skema vedlagt som bilag).

Datahåndtering og opbevaring

Alle spørgeskemaer, der vedrører alle inkludere familier (Baggrundsoplysninger, Interview 1 og Interview 2) vil blive indtastet direkte under interviewene. Rå-kopi af data fra spørgeskemaer opbevares sikret. Der vil blive arbejdet elektronisk i henhold til sikkerhedsforskrifter med anonymiserede kopier af data.
Papiroriginalerne af interview 3 vil blive indtastede og derefter opbevaret i aflåst arkivskab. Rå-kopi af data fra spørgeskemaer opbevares sikret i henhold til Datatilsynets forskrifter. Der vil blive arbejdet elektronisk i henhold til sikkerhedsforskrifter med anonymiseret kopi af data.
Data vil blive behandlet og opbevaret aflåst/utilgængelige og sikkert i fuld overensstemmelse med Datatilsynets Standardvilkår for Forskningsprojekter.
Sponsor, forsøgsansvarlige investigatorer, ph.d.-studerende, statistiker og monitorerende instanser vil kunne få direkte adgang til kildedata.

Styrkeberegning

Studiebase
Hvis man baserer sin power-beregning på et 95%-confidens-intervaller og 90% power, så skal man, for at vise, at BCG vaccinen er associeret 20% beskyttelse mod indlæggelse i første leveår (forekommer blandt 20% af baggrundsbefolkningen) have 3972 (1986 BCG vaccinerede plus 1986 ikke-BCG vaccinerede) børn med i studiet. Hvis man vil påvise 40% beskyttelse mod børneeksem blandt børn under 15 måneder (der forekommer blandt 5 % af baggrundsbefolkningen), skal man have 2115 børn i hver gruppe – BCG vaccinerede og BCG uvaccinerede (Stata beregning). Da alle andre ovennævnte udfald forekommer med hyppigheder mellem 5% til mindst 20% blandt danske børn under to år skulle studiet være i stand til at påvise en klinisk vigtig effekt af BCG vaccination. Svarende til ovennævnte styrkeberegninger som grove mål for det nødvendige antal inkluderede personer i undersøgelsen vil vi stile mod at inkludere 4300 spædbørn. Når sponsor kan opgøre at der er inkluderet 4300 spædbørn, vil de inkluderende afdelinger snarest blive bedt om at stoppe inklusionen.

Blodprøver

For at påvise en klinisk betydningsfuld forskel på 10% i det i blodprøver målte immunrespons med power 90% og $p < 0.05$ behøves der for hver blodprøvetagningsrunde 300 prøver, 150 fra BCG vaccinerede individer og 150 fra individer randomiseret til ikke at være BCG vaccinerede (38). Svarende til ovennævnte styrkeberegninger som grove mål for det nødvendige antal inkluderede spædbørn der får taget blodprøve i undersøgelsen vil vi stile mod ved både opfølgning 1 og opfølgning 2 at tage blodprøver på 300 børn. Når sponsor kan opgøre at der er taget blodprøver på 300 spædbørn, vil de inkluderende afdelinger snarest blive bedt om at stoppe med at tage blodprøver.

Thymusscanning

For at kunne detektere en forskel på 15% i thymusstørrelse mellem de 2 grupper med power 90% og $p < 0.05$ skal 300 børn inkluderes til thymusscanning ved både 3 og 13 måneder (150 BCG-vaccinerede og 150 ikke-BCG-vaccinerede ved hhv. 3 og 13 måneder).

Statistik

Den relative risiko for de definerede udfald vil blive udregnet ved brug af overlevelsesanalyse (Cox regression analyser med resulterende hazard ratios). Børnene vil blive fulgt til de er 15 måneder, svarende til tidspunktet for MFR vaccinationen ifølge det danske vaccinationsprogram. Da studiet er et randomiseret studie forventer vi at mulige confoundere er ligeligt fordelt i de to grupper, men det vil blive undersøgt om nogle af de mulige confoundere herunder om vaccinations teknik/vaccinator påvirker effekten af BCG vaccination på udfaldet, og hvis det er tilfældet vil der blive justeret derfor i analysen. Derudover vil vi undersøge, om effekten af BCG er stærkere i følgende strata:

1. Børn med meget lav fødselsvægt og lav fødselsvægt versus børn med normal fødselsvægt.
2. Børn af forældre med atopisk sygdom versus børn af forældre uden.
3. Ammede børn versus ikke ammede.
4. Børn der vaccineres tidligt (0-2 dage) versus senere.

P-værdier under 0.05 betragtes som statistisk signifikante.

Den mulige effekt af BCG vil bliver analyseret samlet fra 0 til 15 måneder, men også opdelt i perioder svarende til de andre vacciner, der gives i det første leve år:

1. 0-3 mdr hvor der ikke gives andre vacciner.
2. 3-5 måneder, hvor barnet normalt har fået første dosis af di-te-ki-pol-hib + pneu1.

3. 5-12 måneder, hvor barnet normalt har fået anden dosis af di-te-ki-pol-hib + pneu2.
4. 12-15 måneder, hvor barnet normalt har fået tredje doses af di-te-ki-pol-hib + pneu3.

Det formodes, at effekten er størst i de første tre måneder. Ved at analysere i de nævnte perioder vil det være muligt at se, om BCG påvirker responset på de efterfølgende vacciner. De randomiserede studier af BCG i Vestafrika (11;12) har vist at den positive effekt for drenge sker meget tidligt (allerede i den første uge af livet) hvorimod den kommer lidt senere for piger. De mulige effekter og deres timing vil derfor også blive analyseret separat for drenge og piger. Skulle der desuden mens studiet pågår komme ekstraordinære sundhedsproblemer eller sundhedsinterventioner som en særlig kraftig RSV epidemi, influenza epidemi, eller ændringer i Det Danske Børnevaccinationsprogram vil den eventuelle effekt af BCG blive analyseret separat for perioden før og efter begivenheden.

Monitorering og stopperegler

Studiet vil blive monitoreret af GCP enhed og desuden hvert halve år af forskerne i samarbejde med et DSMB. Såfremt BCG har en signifikant negativ effekt ($p<0.01$) eller en signifikant positiv effekt ($p<0.001$) på den generelle sygelighed (hospitalsindlæggelse) eller på den specifikke sygelighed for astma og eksem vil studiet blive stoppet.

Etiske aspekter

Studiet er godkendt af Datatilsynet (J.nr. 2009-41-4141). Studiet er et lægemiddelforsøg og er godkendt af til Lægemiddelstyrelsen, EuDract 2010-021979-85.
Studiet vil blive udført i overensstemmelse med studieprotokollen, myndighedskrav og lovgivning på området.
Studiet kan ikke udføres på dyr, hvis immunforsvar afviger væsentlig fra det menneskelige. Studiet kan ikke udføres på voksne, idet immunforsvaret er særlig påvirkeligt allertidligst i livet og studiet i øvrigt stiler mod at vurdere en beskyttende effekt mod hændelser helt tidligt i livet.

<u>Mulige ulemper for familien og forsøgspersoner</u>

Graviditeten og tidspunktet før og efter en fødsel er en periode i forældrenes liv, hvor man kan forvente at de er følelsesmæssigt meget optagede af og sårbare i forhold til graviditeten og barnet. Moderen kan endvidere være fysisk belastet af graviditeten. At deltage i et klinisk kontrolleret forsøg på dette tidspunkt kan derfor være en belastning for familien.

Alle danske børn får i forvejen en intramuskulær injektion med K-vitamin umiddelbart efter fødslen, og det foreslåede studie vil påføre de spædbørn, der randomiseres til BCG, eet ekstra stik i form af den intradermale BCG vaccination indenfor første leveuge. BCG vaccinen medfører som specificeret nedenfor typisk en lokal-reaktion i huden og desuden et 5 mm i diameter overfladisk hud-ar. Ubehag ved vaccination kan såfremt forældrene ønsker dette imødegås med peroral sukkervand før proceduren.

Ved 3 og 13 måneders alderen planlægges blodprøve på 300 børn. Hver prøve udgør 10 ml, og medfører ikke risiko for barnet. Blodprøvetagning udføres af erfarent sundhedspersonale og eventuelt ubehag kan såfremt forældrene ønsker det imødegås ved brug af lokalbedøvende creme (EMLA) før og sukkervand ved proceduren.

Der ydes ikke vederlag for deltagelse i studiet.

Kontrol-børn i studiet gives ikke placebo-vaccine, da der ikke findes en formulering, der kan fungere som en sand blindet placebo injektion versus BCG vaccinen, der medfører en typisk lokal reaktion og et lille hud-ar.

Undersøgelsen vil give ny og vigtig information vedrørende muligheden for at forbedre alle spædbørns sygelighed, udvikling og dermed livskvalitet.

Forsøgsdeltagerne er raske på vaccinationstidspunktet og blive beskyttet mod tuberkulose. Der er dog ikke tale om et klassisk vaccinationsstudie, da fokus rettes mod andre effektmål end beskyttelse mod den sygdom, vaccinen er rettet imod. Alle børnene er i risiko for at udvikle behandlings- og/eller indlæggelseskrævende sygdom og/eller atopiske sygdomme, hvorfor studiet vurderes at være etisk forsvarligt.

Mulige risici og bivirkninger af BCG vaccination

BCG vaccinen har været så udbredt anvendt i så mange populationer gennem snart 100 år at bivirkningsprofilen er velkendt (se Tabel 1 nedenfor og produktresumé på http://www.produktresume.dk/docushare/dsweb/GetRendition/Document-13163/html). Alvorlige bivirkninger er yderst sjældne og deltagelse i forsøget vurderes derfor kun at medføre en negligeabel risiko, som opvejes af de potentielt klinisk betydningsfulde fordele for deltagerne i form af beskyttelse mod tuberkulose og en potentielt styrket immunforsvar blandt deltagere, der får BCG-vaccine. Man ved, at hyppighed og sværhedsgrad af efterfølgende lokale bivirkninger i form af sårdannelse på indstiksstedet er meget afhængig af injektionsteknikken, idet denne vaccine skal placeres i underhuden (intradermalt), og hvis man sætter vaccinen dybere er der en øget risiko for

sårdannelse. Derfor er en særlig oplæring af alle vaccinatorer påkrævet og planlagt, og desuden vil vaccinationerne blive holdt på så få hænder som muligt.

I et nyligt studie af BCG vaccination umiddelbart efter fødslen blandt knapt 2450 spædbørn fra Guinea Bissau med fødselsvægt < 2500 g observeredes ingen alvorlige bivirkninger (11;12), men derimod en gunstig effekt på sygelighed og dødelighed allerede indenfor de tre første levedøgn.

De potentielt særligt skrøbelige for tidligt fødte børn vil blive fulgt særlig længe med henblik på at se om BCG vaccinen skulle have effekt på børnenes modenhedsgrad ved to-års alderen.

Tabel 1: Bivirkninger ved BCG vaccination.

Ikke almindelig<br>(>1/1000 og <1/100)	Systemisk: Hovedpine, feber.<br>Lokalt: Regional hævelse af lymfeknuder >1 cm.<br>Sårdannelse med væskende sår på injektionsstedet.
Sjælden<br>(<1/1000)	Systemisk: Disseminerede BCG-komplikationer som osteitis eller osteomyelitis. Allergiske reaktioner, herunder anafylaktiske reaktioner.<br>Lokalt: Suppurativ lymfadenitis, abscesdannelse.

Behandling, registrering og rapportering af bivirkninger

Som en del af informationen vedrørende studiet vil forældrene blive skriftligt og mundligt informeret om ovennævnte bivirkninger, herunder blive informeret om, at det er almindeligt og ikke behandlingskrævende, at der efter vaccinationen kommer rødme og hævelse i huden svarende til hvor vaccinen er givet, at der kan komme hævede lymfeknuder i området i op til 4 uger efter vaccinen er givet og at der på længere sigt ofte udvikler sig et lille ca. 5 mm i diameter hud-ar, hvor vaccinen blev givet (svarende til de ”ikke-almindelige bivirkninger” nævnt i skemaet ovenfor). Denne form for bivirkninger vil ikke blive indberettet til Lægemiddelstyrelsen.

Inkluderede børn, der er randomiserede til BCG-vaccination vil blive fulgt for bivirkninger i 4 måneder efter vaccination.

Ved mistanke om bivirkninger udover den ovenfor nævnte vil forældrene blive bedt om at henvende sig telefonisk snarest muligt til den lokale ph.d.-studerende/ansvarshavende investigator med henblik på registrering, rådgivning, og efter behov klinisk kontrol og behandling. Sponser skal

snarest muligt og senest indenfor 5 døgn informeres om "sjældne" bivirkninger, og disse vil blive rapporterede til Lægemiddelstyrelsen i fuld overensstemmelse med Lov om Lægemidler, nr. 1180, § 89 og Bekendtgørelse om kliniske forsøg med lægemidler på mennesker, herunder vil SUSAR (Suspected unexpected serious adverse reactions) blive indberettet til Lægemiddelstyrelsen på dertil indrettet eblanket øjeblikkelig og senest 7 dage efter at bivirkningen er rapporteret til sponsor.

Forsikring

Studiedeltagerne er fuldt forsikrede i henhold til Patientforsikringsloven.

Gevinster og fordele for forsøgsdeltagere

De vaccinerede børn har modtaget en BCG vaccination, der ellers ikke længere gives rutinemæssigt i Danmark, og er dermed specifikt beskyttede mod alvorlig tuberkulose. Flere tidligere studier fra vores og andre grupper peger på at BCG vaccination har en uspecifik positivt stimulerende effekt på immunforsvaret, der medfører generel nedsat sygelighed og reduceret forekomst af immunmedierede sygdomme som astma, eksem og allergi. Disse uspecifikke positive effekter synes stærkest blandt de svageste individer som for eksempel børn med lav fødselsvægt < 2500g.
Risikoen for bivirkninger blandt forsøgsdeltagerne vurderes således opvejet af de forventede positive specifikke og uspecifikke effekter af BCG-vaccinen.

Almen nytte samt videnskabelige fremskridt

Såfremt forsøgsresultaterne viser, at de BCG-vaccinerede spædbørn er mindre syge herunder har mindre atopisk sygdom, bliver indlagt mindre hyppigt og bruger mindre medicin, har dette overordentlig stor samfundsøkonomisk betydning, både via nedsatte udgifter til sundhedsvæsnet og via færre sygedage blandt forældrene. Bedre helbred blandt børn har desuden stor betydning for familierne, en betydning der udover barnet og forældrene omfatter mindst søskende og bedsteforældrene.
Studiets gennemførelse vil medføre et afgørende videnskabeligt fremskridt, da resultaterne af de føromtalte studier i Vestafrika og de tidligere mindre undersøgelser i høj-indkomst-befolkninger, pegende på en positiv immunstimulerende effekt af BCG-vaccinen, ikke umiddelbart kan anføres at gælde generelt før de er efterprøvet i et studie som dette. Så længe man ikke kan anføre, at BCG effekten gælder generelt, kan den mulige positive effekt ikke benyttes systematisk i sundhedsfremmende øjemed.

Inklusion og information (se også Figur 1)

Alle gravide par i optageområderne for Kolding Sygehus, Hvidovre Hospital og Rigshospitalet vil blive identificerede via hospitalernes svangreambulatorier. Disse par udgør studiebasen og vil blive registrerede i studiedatabase (e-crf) som mulige forsøgsdeltagere og få tilsendt brev med information om studiet, forespørgsel om deltagelse og samtykkeerklæringer (Vedlagt som bilag).
To til fire uger efter fremsendelsen af brevet vil alle inviterede blive kontaktet telefonisk af sundhedsfagligt uddannet personale fra forskningsgruppen, som på hvert af de tre hospitaler er ansvarlig for inklusionen af deltagere, og spurgt, om de ønsker at deltage i studiet. Interesserede forældre vil blive registrerede med navn, adresse, e-mail og telefonnummer og spurgt om de helst 1) vil modtage mundtlig information om studiet med det samme, 2) vil ringes op på et andet tidspunkt der passer familien, således at telefonsamtalen kan foregå uforstyrret eller 3) om de foretrækker at blive yderligere informerede om studiet ved et informationsmøde på deres forventede fødested. Idet man som gravid ofte kan være tryghedssøgende og derfor har det bedst i sin egen "rede", og desuden kan være tung, træt og besværet, hvilket kan gøre alle logistiske manøvrer til en pestilens, kan den gravide og partneren således selv vælge rammer og afgøre, om de foretrækker at kunne være hjemme i trygge rammer til informationssamtalen. Det skal her understreges at forskergruppen fuldt forstår og ønsker at leve op til Videnskabsetisk Komites krav om at informationssamtalen foregår uforstyrret og hvis det ønskes med bisidder, men vores forskergruppe mener bestemt, at man også godt telefonisk kan sikre at disse forhold er på plads. Studiedeltagelse noteres i vandrejournalen af forældrene og i hospitalets journal. I det tilsendte skriftlige materiale er forældrene blevet gjort opmærksomme på, at de efter inklusion til enhver tid kan trække sig ud af studiet, uden at det vil få nogen konsekvenser for behandlingen af deres barn. Desuden vil tegn til bivirkninger efter vaccinen være nævnt, og det vil fremgå at forældrene bedes søge læge ved tegn hertil. Den skriftlige deltager-information vil omfatte Den Centrale Videnskabsetiske Komites fortrykte ”Forsøgspersoners rettigheder i et biomedicinsk forskningsprojekt”, Samtykkeerklæring og Fuldmagtserklæring (Vedlagt som bilag). I den skriftlige information anvises en hjemmeside, der i 2 år efter forsøgets afslutning vil oplyse forældre om mulige uventede risici, studiets forløb og resultater. Alle disse oplysninger vil blive gentaget under informationssamtalen.
Forældre, som foretrækker at mødes, eller forældre der er i tvivl om de ønsker at deltage i studiet, vil mødes efter aftale med et medlem af forskningsgruppen.

Ved både den telefoniske kontakt og et evt. møde vil informanten først sikre sig at der virkelig er ro og tid til samtalen, ved at angive at det er en forudsætning for en god samtale at samtalen kan foregå uforstyrret og uden afbrydelse, og at der er en bisidder tilstede hvis det ønskes. Herefter gives information om studiet i henhold til Videnskabsetisk Komites retningslinjer, og sluttelig svares på forældrenes spørgsmål. Informanten vil elektronisk registrere, om forældrene er interesserede i deltagelse, med interesserede forældre lave et kort interview til indsamling af baggrundsoplysninger (Vedlagt som bilag) og bede forældrene om, efter mindst et døgns betænkningstid men helst indenfor en uge, at indsende underskrevne samtykkeerklæringer fra begge forældre. Interviewet med baggrundsoplysninger vil kun blive brugt som data i studiet, såfremt der foreligger underskrevne samtykkeerklæringer.

Svangreambulatorierne vil modtage information om alle de gravide par, der er interesserede i at deltage i forsøget, så jordemødrene er orienterede om dette ved fødslen. Såfremt der ikke skulle foreligge underskrevne samtykkeerklæringer når spædbørnene skal randomiseres til +/- BCG-vaccine efter fødslen, sikres det at samtykkeerklæringer underskrives inden randomisering.

Alle forældre i Kolding, også de, der oplyser, at de ikke ønsker at deltage i vaccinationsstudiet, samt de forældre, der endnu ikke har besluttet sig, vil blive anmodet om at svare på nogle få spørgsmål om det at skulle tage beslutning om, om ens nyfødte skal BCG vaccineres eller ej. Såfremt de accepterer, vil der blive stillet nogle spørgsmål, der bygger på validerede spørgsmål, der bl.a. har været anvendt i forbindelse med det at skulle tage stilling til H1N1 vaccinationer. Desuden vil der blive foretaget et mindre forstudium for at sikre, at spørgsmålene stilles forståeligt. Spørgsmålene vil være helt neutrale og på ingen måde hverken lægge pres på de vordende forældre eller indikere, at deres beslutning ikke er den rigtige (Vedlægges som bilag). Vi finder det væsentligt, at det strukturerede interview bliver af så høj kvalitet som muligt, hvorfor der planlægges pilottestning heraf, således at der vil kunne komme mindre formuleringsmæssige ændringer efter testningen.

Sundhedsfagligt uddannet personale fra forskningsgruppen vil indsamle, elektronisk registrere og aflåst arkivere underskrevne Samtykkeerklæringer fra begge forældre. Den første kontakt med mulige deltagere vil således være skriftlig og derefter personlig og mundtlig med mulighed for uddybende spørgsmål med god tid til overvejelse og beslutning. Forældrene vil således selv kunne vælge om de foretrækker at modtage mundtlig information telefonisk, hjemme i trygge omgivelser uden besvær med transport, eller ved et uforstyrret møde på Børneafdelingen.

Vaccination

På hvert hospital vil en gruppe personer på vaccinationskursus med diplom blive oplært i den korrekte vaccinationsteknik. Afhængig af logistikken på de enkelte hospitaler, vil børnene enten blive vaccinerede på fødegangen i timerne efter fødslen, på barselsafdelingerne i døgnene efter fødslen, ved de obligatoriske blodprøver til neonatal screening via PKU kort cirka 2 døgn efter fødslen eller ved de obligatoriske hørescreeninger cirka 5 døgn efter fødslen.

Randomisering

Børn af forældre, der indvilliger i at deltage, vil blive elektronisk randomiserede til BCG vaccine eller ingen vaccination. Såfremt forældre til børn, der er randomiserede til ingen vaccination alligevel ønsker BCG vaccination af deres barn, vil disse forældre blive opfordret til at få barnet vaccineret hos egen læge og børnene vil udgå af studiet.

Biobank

Overskydende blod fra blodprøverne ved 3 og 13 måneders alderen vil opbevares i biobank til senere analyser. Prøverne vil være opbevaret i anonymiseret form og fremtidige analyser forudsætter godkendelse fra Videnskabsetisk Komite.

Studiegruppe

Studiet er initieret af 1. reservelæge Lone Graff Stensballe og prof. Peter Aaby.

Rigshospitalet

På Rigshospitalet vil prof. Gorm Greisen fra neonatalafdeling GN og Lone Graff Stensballe fra De Pædiatriske Klinikker være overordnet ansvarshavende for og supervisere studieforløbet.

Lone Graff Stensballe er studiets sponser og desuden forsøgsansvarlig investigator på Rigshospitalet.

Lone Graff Stensballe, 1. reservelæge, PhD.
BørneUngeKlinikken 4072, Juliane Marie Centret, Rigshospitalet.
Blegdamsvej 9
DK- 2100 København Ø
Tlf.: 60 22 80 92
Email: lone.graff.stensballe@rh regionh.dk

Hvidovre Hospital

På Hvidovre Hospital vil prof. Ole Pryds fra Børneafdelingen være forsøgsansvarlig investigator og supervisere studieforløbet.

Ole Pryds, prof, dr.med., neonatolog.
Børneafdelingen, Hvidovre Hospital.
Kettegårds Allé 30
2650 Hvidovre
Tlf.: 36323632
Email: pryds@dadlnet.dk

Kolding Sygehus

På Kolding Sygehus vil overlæge Poul-Erik Kofoed være forsøgsansvarlig investigator og supervisere studieforløbet.

Poul-Erik Kofoed, ledende overlæge, dr.med., børnelæge.
Børneafdelingen, Kolding Sygehus.

Skovvangen 2-8
6000 Kolding
Tlf.: 76362000
Email: Poul.Erik.Kofoed@slb.regionsyddanmark.dk

Statens Serum Institut

Statens Serum Institut producerer den i studiet anvendte BCG vaccine og har markedsføringstilladelsen. Statens Serum Institut (direktør Niels Strandberg og medical affairs manager Mie Hermansen Kusk) er i detaljer informeret om studiet, men er herudover som institution ikke involveret i studiet, herunder ikke økonomisk involveret.

Prof. Peter Aaby og seniorforsker Christine Benn er epidemiologiske rådgivere.
Seniorstatistiker Henrik Ravn medvirker som overordnet rådgivende statistiker.
Fælles arbejdsadresse:
Forskningscenter for Vitaminer og Vacciner (CVIVA)
Bandim Health Project
Bygn. 206
Artillerivej 5
DK-2300 København S
Tlf.: 32683268
Email:
p.aaby@bandim.org
cb@ssi.dk
hjn@ssi.dk

Statens Serum Institut vil være ansvarlig for at analysere blodprøver taget ved 13 måneders alderen for antistoffer mod difteri og tetanus.

Publikation

Studiet anmeldes til www.ClinicalTrials.Gov. Positive såvel som negative studiefund publiceres. Som alternativ offentliggørelse vil studieresultaterne desuden blive opsummeret på studiets hjemmeside, om muligt med link til de relevante publikationer.
Det er planlagt at hvert deltagende hospital vil lave tre til seks publikationer omhandlende deres fokusområde (se nedenfor) af det samlede studium. Alle publikationer udgående fra studiet vil blive stilet mod internationale peer-reviewed medicinske tidsskrifter med størst mulig impact.

Rigshospitalet

På publikationerne fra Rigshospitalet vil Lone Graff Stensballe være anden-forfatter og prof. Gorm Greisen være sidsteforfatter. Rigshospitalets fokusområde vil være vækst, udvikling, morbiditet, for tidligt fødte spædbørn og spædbørn med lav fødselsvægt.

Hvidovre Hospital

På publikationerne fra Hvidovre Hospital vil prof. Ole Pryds eller Overlæge Dorthe Jeppsen fra Børneafdelingen være sidsteforfatter. Hvidovre Hospitals fokusområde vil være immunologiske aspekter målt paraklinisk i blodprøver og ved thymusscanninger.

På Kolding Sygehus

På publikationerne fra Kolding Sygehus vil overlæge Poul-Erik Kofoed eller en anden hovedvejleder være sidsteforfatter. Kolding Sygehus fokusområde vil være de atopiske sygdomme målt som forekomst af astma og eksem og desuden at undersøge forældres holdning til og accept af BCG vaccination af deres nyfødte barn, samt om dette har indflydelse på senere accept af andre vaccinationer i det danske børnevaccinationsprogram.

Sammenfattende publikation

På den publikation, der sammenfatter resultaterne ved at poole resultaterne fra alle de inkluderede patienter, vil Lone Graff Stensballe være førsteforfatter, epidemiolog Christine Stabell Benn og senior-statistiker Henrik Ravn være medforfattere og professor Peter Aaby være sidsteforfatter.

Økonomi

Studiet er planlagt og initieret af Lone Graff Stensballe og Peter Aaby.

Studiet er af Grundforskningsfonden finansieret via det nyopstartede Grundforskningscenter for Vitaminer og Vacciner (CVIVA) som er ledet af Christine Stabell Benn. Restfinansiering søges via ikke-kommercielle fondsmidler. I tilfælde af økonomisk tilknytning mellem sponsor/forsøgsansvarlig og støttegiver (”conflict of interest”) vil dette blive anført på publikationer samt beskrevet på studiet hjemmeside.

Der vil ikke blive betalt vederlag for deltagelse.

Referencer

(1) Roth A, Jensen H, Garly ML, Djana Q, Martins CL, Sodemann M, et al. Low birth weight infants and Calmette-Guerin bacillus vaccination at birth: community study from Guinea-Bissau. Pediatr Infect Dis J 2004 Jun;23(6):544-50.

(2) Aaby P, Jensen H, Gomes J, Fernandes M, Lisse IM. The introduction of diphtheria-tetanus-pertussis vaccine and child mortality in rural Guinea-Bissau: an observational study. Int J Epidemiol 2004 Apr;33(2):374-80.

(3) Aaby P, Jensen H, Garly ML, Bale C, Martins C, Lisse I. Routine vaccinations and child survival in a war situation with high mortality: effect of gender. Vaccine 2002 Nov 22;21(1-2):15-20.

(4) Kristensen I, Aaby P, Jensen H. Routine vaccinations and child survival: follow up study in guinea- bissau, west africa [In Process Citation]. BMJ 2000 Dec 9;321(7274):1435-9.

(5) Aaby P, Vessari H, Nielsen J, Maleta K, Benn CS, Jensen H, et al. Sex differential effects of routine immunizations and childhood survival in rural Malawi. Pediatr Infect Dis J 2006 Aug;25(8):721-7.

(6) Velema JP, Alihonou EM, Gandaho T, Hounye FH. Childhood mortality among users and non-users of primary health care in a rural west African community. Int J Epidemiol 1991 Jun;20(2):474-9.

(7) Garly ML, Martins CL, Balé C, Baldé MA, Hedegaard KL, Gustafson P, et al. BCG scar and positive tuberculin reaction associated with reduced child mortality in West Africa: A non-specific beneficial effect of BCG? Vaccine 2003;21:2782-90.

(8) Roth A, Gustafson P, Nhaga A, Djana Q, Poulsen A, Garly ML, et al. BCG vaccination scar associated with better childhood survival in Guinea-Bissau. Int J Epidemiol 2005 Jun;34(3):540-7.

(9) Roth A, Sodemann M, Jensen H, Poulsen A, Gustafson P, Weise C, et al. Tuberculin reaction, BCG scar, and lower female mortality. Epidemiology 2006 Sep;17(5):562-8.

(10) Aaby P, Shaheen SO, Heyes CB, Goudiaby A, Hall AJ, Shiell AW, et al. Early BCG vaccination and reduction in atopy in Guinea-Bissau. Clin Exp Allergy 2000 May;30(5):644-50.

(11) Aaby P, Roth A, Ravn H, Napirna BM, Rodrigues A, Lisse IM, et al. Randomized trial of BCG vaccination at birth to low-birth-weight children: beneficial nonspecific effects in the neonatal period? J Infect Dis 2011 Jul 15;204(2):245-52.

(12) Biering-Sorensen S, Aaby P, Napirna BM, Roth A, Ravn H, Rodrigues A, et al. Small randomized trial among low-birth-weight children receiving bacillus Calmette-Guerin vaccination at first health center contact. Pediatr Infect Dis J 2012 Mar;31(3):306-8.

(13) Strachan DP. Family size, infection and atopy: the first decade of the "hygiene hypothesis". Thorax 2000 Aug;55 Suppl 1:S2-10.

(14) Marchant A, Goetghebuer T, Ota MO, Wolfe I, Ceesay SJ, De Groote D, et al. Newborns develop a Th1-type immune response to Mycobacterium bovis bacillus Calmette-Guerin vaccination. J Immunol 1999 Aug 15;163(4):2249-55.

(15) Prescott SL, Macaubas C, Smallacombe T, Holt BJ, Sly PD, Holt PG. Development of allergen-specific T-cell memory in atopic and normal children [see comments]. Lancet 1999 Jan 16;353(9148):196-200.

(16) Eifan AO, Akkoc T, Ozdemir C, Bahceciler NN, Barlan IB. No association between tuberculin skin test and atopy in a bacillus Calmette-Guerin vaccinated birth cohort. Pediatr Allergy Immunol 2009 Sep;20(6):545-50.

(17) Dilli D, Bostanci I, Dallar Y. Do different vaccination regimens for BCG and hepatitis B affect the development of allergic disorders in early childhood? J Asthma 2008 Mar;45(2):155-9.

(18) Soysal A, Bahceciler N, Barlan I, Bakir M. Lack of an inverse association between tuberculosis infection and atopy: by T-cell-based immune assay (RD1-ELISpot). Pediatr Allergy Immunol 2008 Dec;19(8):709-15.

(19) Li J, Zhou Z, An J, Zhang C, Sun B, Zhong N. Absence of relationships between tuberculin responses and development of adult asthma with rhinitis and atopy. Chest 2008 Jan;133(1):100-6.

(20) Ahmadiafshar A, Parchegani MR, Moosavinasab N, Koosha A. A Study of Relation between BCG Scar and Atopy in Schoolchildren of Zanjan City. Iran J Allergy Asthma Immunol 2005 Dec;4(4):185-8.

(21) Omar MT, Ahmed SH, Sarkis NN. A study of the association between delayed type hypersensitivity reaction to mycobacterium tuberculosis and atopy in asthmatic children. J Egypt Public Health Assoc 2005;80(3-4):463-74.

(22) Obihara CC, Beyers N, Gie RP, Potter PC, Marais BJ, Lombard CJ, et al. Inverse association between Mycobacterium tuberculosis infection and atopic rhinitis in children. Allergy 2005 Sep;60(9):1121-5.

(23) Annus T, Montgomery SM, Riikjarv MA, Bjorksten B. Atopic disorders among Estonian schoolchildren in relation to tuberculin reactivity and the age at BCG vaccination. Allergy 2004 Oct;59(10):1068-73.

(24) da Cunha SS, Cruz AA, Dourado I, Barreto ML, Ferreira LD, Rodrigues LC. Lower prevalence of reported asthma in adolescents with symptoms of rhinitis that received neonatal BCG. Allergy 2004 Aug;59(8):857-62.

(25) Townley RG, Barlan IB, Patino C, Vichyanond P, Minervini MC, Simasathien T, et al. The effect of BCG vaccine at birth on the development of atopy or allergic disease in young children. Ann Allergy Asthma Immunol 2004 Mar;92(3):350-5.

(26) Bager P, Rostgaard K, Nielsen NM, Melbye M, Westergaard T. Age at bacille Calmette-Guerin vaccination and risk of allergy and asthma. Clin Exp Allergy 2003 Nov;33(11):1512-7.

(27) Ota MO, Van Der Sande MA, Walraven GE, Jeffries D, Nyan OA, Marchant A, et al. Absence of association between delayed type hypersensitivity to tuberculin and atopy in children in The Gambia. Clin Exp Allergy 2003 Jun;33(6):731-6.

(28) Marks GB, Ng K, Zhou J, Toelle BG, Xuan W, Belousova EG, et al. The effect of neonatal BCG vaccination on atopy and asthma at age 7 to 14 years: an historical cohort study in a community with a very low prevalence of tuberculosis infection and a high prevalence of atopic disease. J Allergy Clin Immunol 2003 Mar;111(3):541-9.

(29) Krause TG, Hviid A, Koch A, Friborg J, Hjuler T, Wohlfahrt J, et al. BCG vaccination and risk of atopy. JAMA 2003 Feb 26;289(8):1012-5.

(30) Jang AS, Son MH. The association of airway hyperresponsiveness and tuberculin responses. Allergy 2002 Apr;57(4):341-5.

(31) Wong GW, Hui DS, Tam CM, Chan HH, Fok TF, Chan-Yeung M, et al. Asthma, atopy and tuberculin responses in Chinese schoolchildren in Hong Kong. Thorax 2001 Oct;56(10):770-3.

(32) Gruber C, Kulig M, Bergmann R, Guggenmoos-Holzmann I, Wahn U. Delayed hypersensitivity to tuberculin, total immunoglobulin E, specific sensitization, and atopic manifestation in longitudinally followed early Bacille Calmette-Guerin-vaccinated and nonvaccinated children. Pediatrics 2001 Mar;107(3):E36.

(33) Yilmaz M, Bingol G, Altintas D, Kendirli SG. Correlation between atopic diseases and tuberculin responses. Allergy 2000 Jul;55(7):664-7.

(34) Steenhuis TJ, van Aalderen WM, Bloksma N, Nijkamp FP, van der LJ, van LH, et al. Bacille-Calmette-Guerin vaccination and the development of allergic disease in children: a randomized, prospective, single-blind study. Clin Exp Allergy 2008 Jan;38(1):79-85.

(35) Andersen TF, Madsen M, Jorgensen J, Mellemkjoer L, Olsen JH. The Danish National Hospital Register. A valuable source of data for modern health sciences. Dan Med Bull 1999 Jun;46(3):263-8.

(36) Hasselbalch H, Jeppesen DL, Engelmann MD, Michaelsen KF, Nielsen MB. Decreased thymus size in formula-fed infants compared with breastfed infants. Acta Paediatr 1996 Sep;85(9):1029-32.

(37) Heilmann C, Grandjean P, Weihe P, Nielsen F, Budtz-Jorgensen E. Reduced antibody responses to vaccinations in children exposed to polychlorinated biphenyls. PLoS Med 2006 Aug;3(8):e311.

(38) Ramsay ME, Rao M, Begg NT, Redhead K, Attwell AM. Antibody response to accelerated immunisation with diphtheria, tetanus, pertussis vaccine. Lancet 1993 Jul 24;342(8865):203-5.

Figur 1. Inklusionsprocedure for studiet "BCG vaccination og sygelighed blandt danske spædbørn".

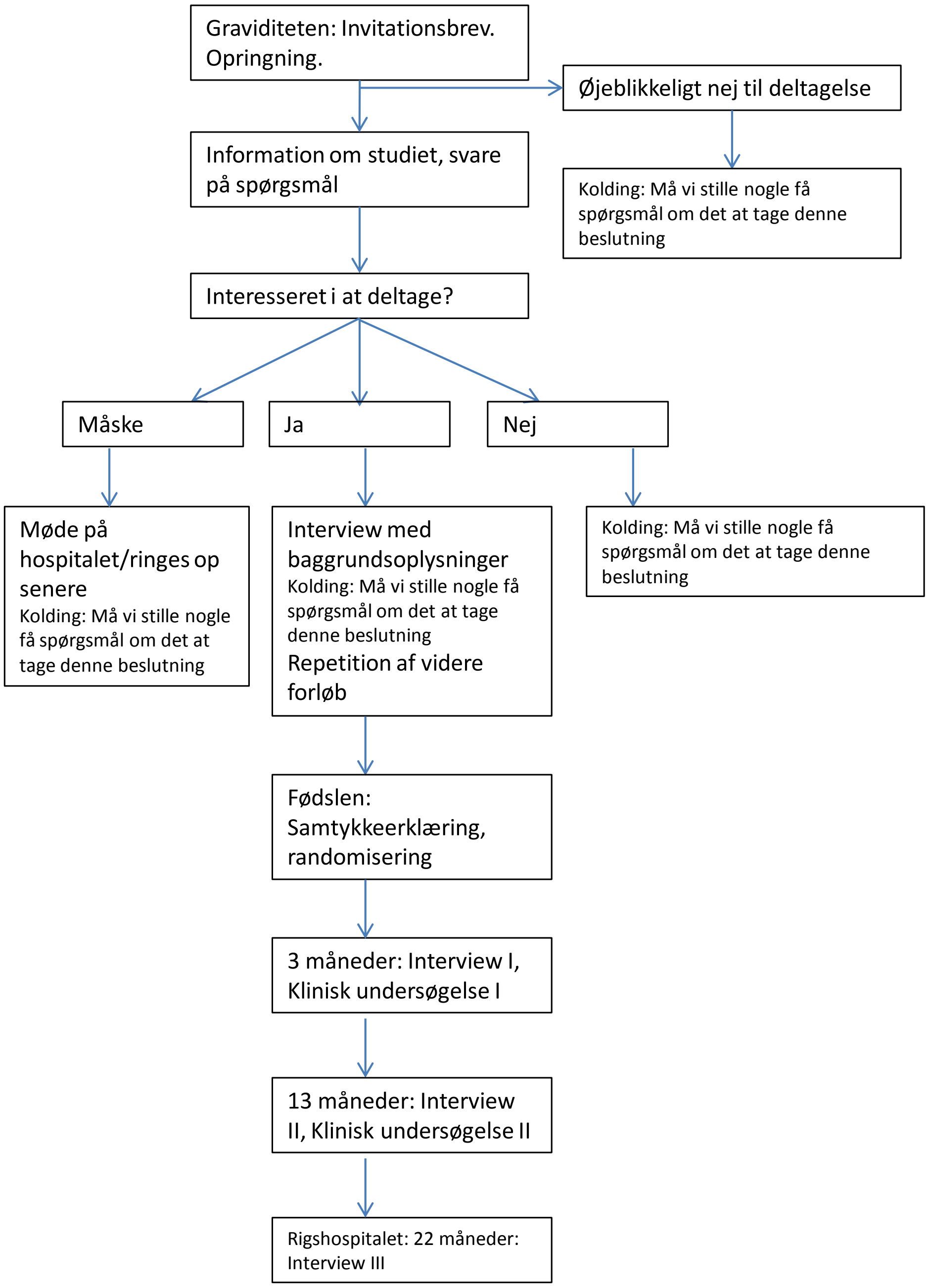

Figur 2. Tidslinje for studiet ”BCG vaccination og sygelighed blandt danske spædbørn”.

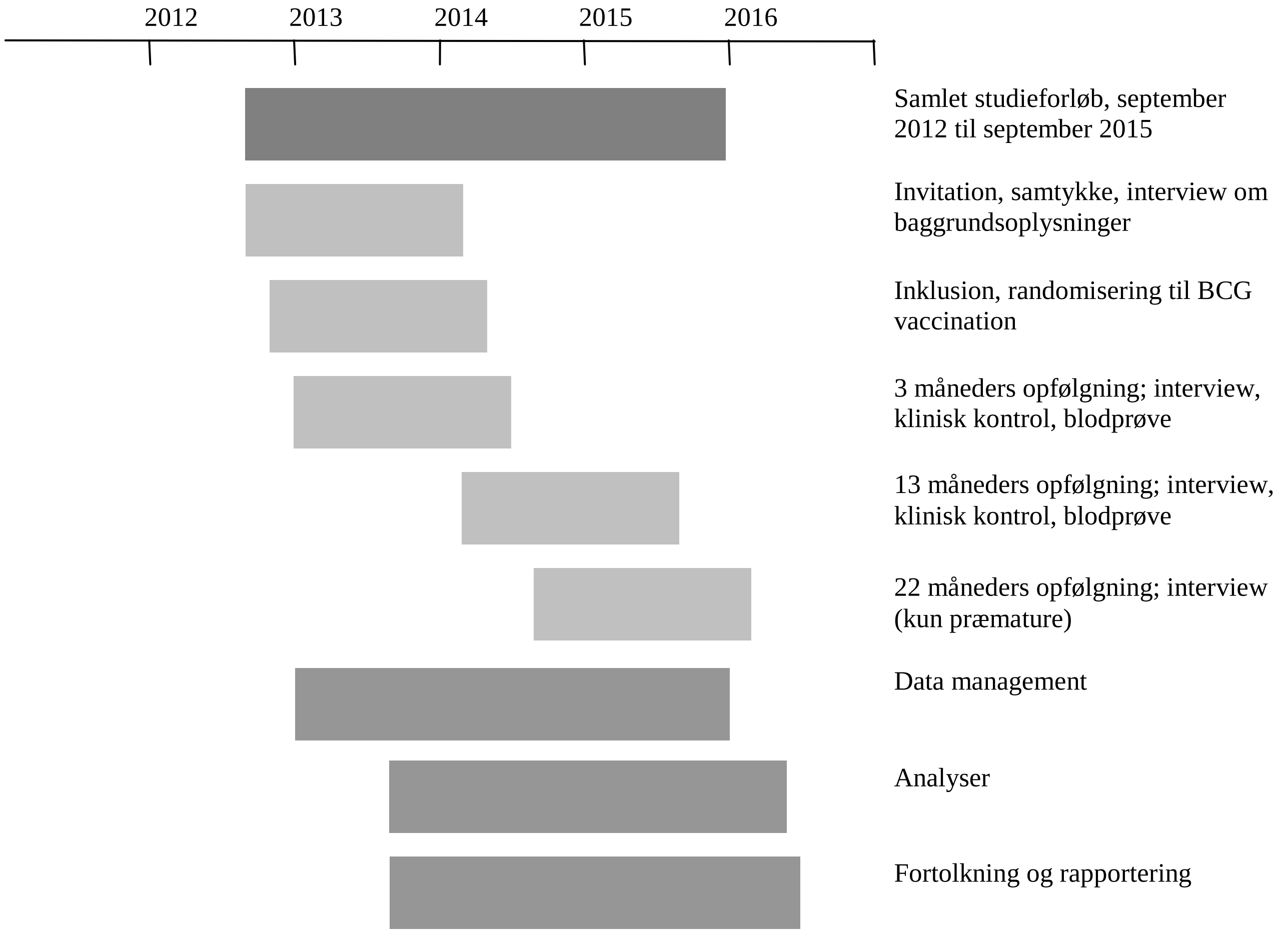

Figur 3. Tidslinje for deltagere i studiet ”BCG vaccination og sygelighed blandt danske spædbørn”.

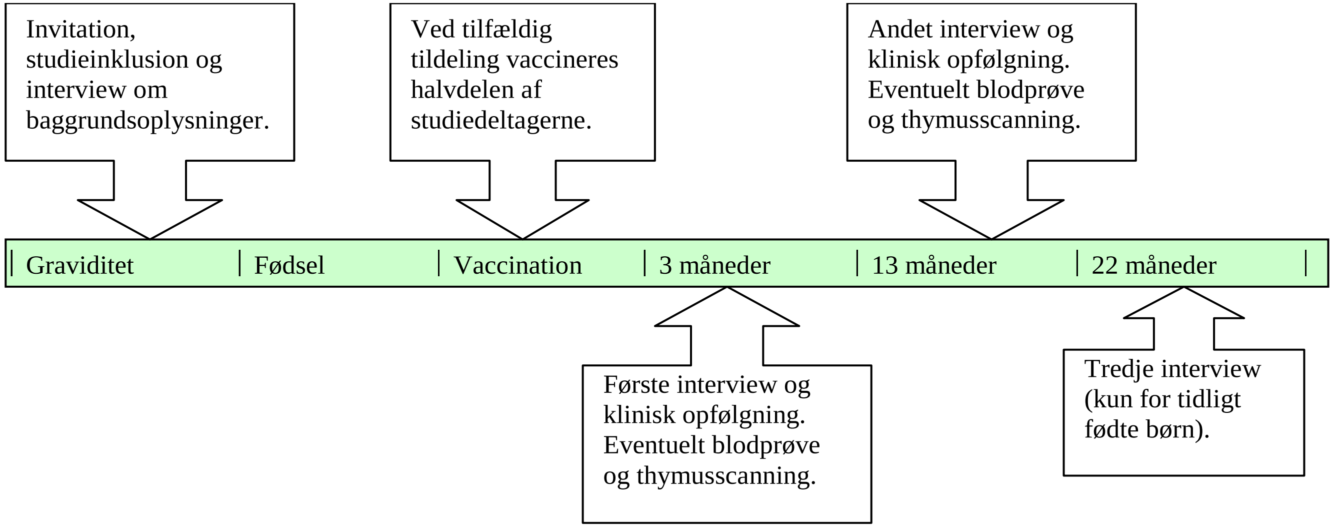

Supplement: S3 File — (PDF) [file pone.0154541.s004.pdf]
