# Supplementary material for: Lack of a Negative Effect of BCG-Vaccination on Child Psychomotor Development: Results from the Danish Calmette Study - A Randomised Clinical Trial
Source: PLoS One. 2016 Apr 28;11(4):e0154541. doi: 10.1371/journal.pone.0154541 (PMC4849633; doi:10.1371/journal.pone.0154541)
Supplement: S4 File — (DOCX) [file pone.0154541.s005.docx]

Serial number: "Address Block" (S5)

**Consent of the custodial parent to their child's participation in health science research.**

Research Project Title: *Calmette-vaccination and morbidity among Danish toddlers*

Statement by the parent with custody:

I / we have received written and oral information and I / we know enough about the objectives, methods, advantages and disadvantages to give our consent.

I / we know that it is voluntary and that I / we can always withdraw our consent without my / our daughter / son loses his present or future rights to treatment.

I / we consent to the child of ________________________________________ (mother's name) will participate in the research project. I / we have received a copy of this consent form and a copy of the written information on the project for personal use.

The name or names of the custodial parent (s):

____________________________________ ______________________________________

Date: _____________ Signature: ________________________________________________

Date: _____________ Signature: ________________________________________________

Do you want to be informed about the research project results as well as possible implications for

your child ?:

Yes ____ (x) No _____ (x)

**Declaration of the person who supplied the information for the parent(s):**

I declare that the parents have received oral and written information about the trial and have had the opportunity to question to me further.

In my view, given sufficient information has been given to allow the parents to decide on the child's participation in the trial.

The name of the person who supplied the information:

Date: _______________ Signature: _ _________________

**Declaration by the primary investigator:**

I declare that the parent / child has received oral and written information about the trial.

In my view, sufficient information has been given to allow parents to decide on the child's participation in the trial.

The name of the primary investigator: Lone Graff Stensballe

Date: _______________ Signature: _________________________

Project Identification: EudraCT 2010-021979-85
