# Supplementary material for: Lack of a Negative Effect of BCG-Vaccination on Child Psychomotor Development: Results from the Danish Calmette Study - A Randomised Clinical Trial
Source: PLoS One. 2016 Apr 28;11(4):e0154541. doi: 10.1371/journal.pone.0154541 (PMC4849633; doi:10.1371/journal.pone.0154541)
Supplement: S1 Text — (DOCX) [file pone.0154541.s008.docx]

Supplementary Text 1: STATA code used for statistical analyses in this manuscript.

/* Do-file containing statistical analyses for manuscript

"Non-specific effects of BCG-vaccination on child psychomotor development:

results from The Danish Calmette Study - a randomised clinical trial" */

cd H:\forsk\csdevelopment

use bcgdevelopment, clear

/////////////////

/// Analyses ///

///////////////

//////////////////////

// Primary outcome //

////////////////////

// Age-adjusted sum score of ASQ at 13 months //

// (age-adjustment in the form of age as a predictor variable)

regress asqs b2.rtreat b2.premature asqage

bysort rtreat: summ asqs, d

summ asqs, d

// Sensitivity adjusting for randomization imbalances

merge m:1 rid using H:\forsk\csdata\acinkl, nogen

drop inkl_sys_date - BCG_inkl03x BCG_inkl05 - BCG_inkl07 BCG_inkl09a - BCG_inkl18

replace BCG_inkl04 = 3 if BCG_inkl04 >= .

replace BCG_inkl08 = 3 if BCG_inkl08 >= .

regress asqs b2.rtreat b2.premature asqage b2.BCG_inkl04 b2.BCG_inkl08

// Subgroup analyses: Prematurity, Sex, BCG-immunisation within two days of birth.

// Prematurity (GA < 37 wks)

bysort premature rtreat: summ asqs, d

bysort premature: summ asqs, d

regress asqs b2.rtreat#i.premature b2.premature asqage

contrast rtreat#premature

misschk asqs rtreat premature asqage if premature == 1

misschk asqs rtreat premature asqage if premature == 2

// --> OBS: Try adjuting for gestational age in days, since it seems the controls were older

hist gadage, by(premature rtreat)

regress asqs b2.rtreat asqa gadage if premature == 1

// Sex

bysort sex rtreat: summ asqs, d

bysort sex: summ asqs, d

regress asqs b2.rtreat#i.sex i.sex b2.premature asqage

contrast rtreat#sex

misschk asqs rtreat sex asqage if sex == 1

misschk asqs rtreat sex asqage if sex == 2

// by bcg within 2 days /// Mail fra LGS:

// Early vaccination

g earlybcg=1 if vage<=2 & PPrtreat==1

replace earlybcg=2 if vage>2 & PPrtreat==1

replace earlybcg=0 if PPrtreat==2

tab earlybcg, m

misschk asqs earlybcg asqage if earlybcg == 1

misschk asqs earlybcg asqage if earlybcg == 2

bysort earlybcg: summ asqs, d

regress asqs i.earlybcg b2.premature asqage

contrast earlybcg

// Secondary outcomes //

// Sub-scale scores (communication, fine motor, gross motor,

// problem solving, personal and social development) at 13 months in mature infants

gen asqresponder = .

replace asqresp = 1 if anymiss == 0

replace asqresp = 2 if anymiss > 0 & anymiss < .

replace asqresp = 3 if anymiss == .

label define asqresponder 1 "Complete responder" 2 "Partial responder" ///

3 "Non-responder", replace

label value asqresponder asqresponder

tab asqr rtreat

tabulate rtreat if asqres == 1, sum(coms)

regress coms b2.rtreat b2.premature asqage if asqres == 1

tabulate rtreat if asqres == 1, sum(fms)

regress fms b2.rtreat b2.premature asqage if asqres == 1

// The fms distribution is not normal:

ranksum fms if asqres == 1, by(rtreat)

regress gms b2.rtreat b2.premature asqage if asqres == 1

tabulate rtreat if asqres == 1, sum(gms)

regress pss b2.rtreat b2.premature asqage if asqres == 1

tabulate rtreat if asqres == 1, sum(pss)

regress psds b2.rtreat b2.premature asqage if asqres == 1

tabulate rtreat if asqres == 1, sum(psds)

///

// Per protocol analysis

///

regress asqs b2.PPrtreat b2.premature asqage

regress coms b2.PPrtreat b2.premature asqage

regress fms b2.PPrtreat b2.premature asqage

regress gms b2.PPrtreat b2.premature asqage

regress pss b2.PPrtreat b2.premature asqage

regress psds b2.PPrtreat b2.premature asqage

//////////////////////////

///// Tables //////////

////////////////////

use bcgdevelopment, clear

gen asqresponder = .

replace asqresp = 1 if anymiss == 0

replace asqresp = 2 if anymiss < 10 & anymiss > 0

replace asqresp = 3 if anymiss > 9

gen asqrbinary = 1 if asqres == 1

replace asqrbinary = 2 if asqres !=1

lab var asqrbin "binary asqresponder, 1= responder, 2 = partial and non-resp"

gen matagedays = barndob - mordob

gen matageyrsdeci = matagedays/365.25

gen matageyrs = int(matageyrsdeci)

// Maternal age at birth, mean (SD)

tab rtreat asqrbin, sum(matageyrs)

drop _merge

merge m:1 rid using H:\forsk\csdata\acinkl

drop if _merge == 2

drop _merge

drop inkl_sys_date - BCG_inkl04b BCG_inkl09a - BCG_inkl14b BCG_inkl16_1a - BCG_inkl18

// Keeping mothers education, parental smoking during pregnancy, older biological siblings

// Mothers level of education at inclusion

recode BCG_inkl03 (1 2 3 = 1) (4 5 = 2) (6 7 = 3) (8 . .a = 4), gen(matedu)

label define matedu 1 "Basic schooling and non-theoretical education" ///

2 "Theoretical education incl BA level" 3 "Master level or more" ///

4 "Unknown"

label values matedu matedu

bysort asqrbin: tab matedu rtreat, m col

tab matedu asqrbin if premature == 2, m col chi2

// Maternal smoking during pregnancy

bysort asqrbin: tab BCG_inkl06 rtreat, m col

// Gestational age, median (10/90 decile range or IQR)

bysort asqresp rtreat: summ gadage, d

// Prematurity:

bysort asqrbin: tab premature rtreat, col

// Child age at developmental follow-up, median (IQR)

bysort asqrbin rtreat: summ asqage, d

// Child sex

bysort asqrbin: tab sex rtreat, m col

// Older biological siblings at inclusion

merge 1:1 ridbarn using H:\forsk\csdata\actele2

drop if _merge == 2

drop _merge

drop tele2_sys_date - BCG_fub16bcbb BCG_fub17a - BCG_fub19 BCG_fub21 - BCG_fubkomm

gen oldsib = .

replace oldsib = 1 if BCG_inkl15 >= 1 & BCG_inkl15 < .

replace oldsib = 0 if BCG_inkl15 < 1

tab oldsib asqrbin, col m chi2

tab matedu asqrbin, col m chi2

bysort asqrbin: tab oldsib rtreat, m col

// Exclusively breastfed at three month follow-up

merge 1:1 ridbarn using H:\forsk\csdata\actele1

drop if _merge == 2

drop _merge

drop tele1_sys_date - BCG_fu15a BCG_fu16e - BCG_fukomm

bysort asqrbin: tab BCG_fu16 rtreat, m col

// Multiple birth

bysort asqrbin: tab fold rtreat, m col

///////////////////////

// Results section ///

/////////////////////

tab asqrbin rtreat, col chi2

bysort asqrbin: tab matedu

disp 809 - 212 // 597

disp 3453 - 695 // 2758

tabi 212 597 \ 695 2758, chi2

bysort asqrbin: tab oldsib

tab asqrbin oldsib, chi2

* ASQ score for complete questionnaires:

bysort rtreat: summ asqs

* Age at follow-up

bysort rtreat: summ asqage if asqs <., d

* Equal distribution of responders between allocation groups?

tab asqrbin rtreat, chi2 col

* Equal distribution of matedu and siblings between allocation groups for non/partial resp?

tab matedu asqrbin, col m chi2

tab oldsib asqrbin, col m chi2

////////////////////////////////

////// Figures /¤/¤/¤/¤/¤/¤

////////////////////////////////

// Supplementary figure A: ASQ score by allocation group and age at follow-up

egen asqacat3 = cut(asqage), group(6) label

graph box asqs, by(asqacat3) over(rtreat)

g treat = 1 if rtreat == 2

replace treat = 2 if rtreat == 1

label define treat 1 "Control" 2 "BCG"

label values treat treat

label variable asqs "Total ASQ Score"

label define boxplot 0 "302 - 365" 1 "366 - 371" 2 "372 - 376" ///

3 "377 - 386" 4 "387 - 410" 5 "411 - 663"

label values asqacat3 boxplot

graph box asqs, over(treat) over(asqacat3) asyvars scheme(s1mono) ///

b1title(Age at follow-up in days) name(asqage, replace)

hist asqs, by(treat, note(.)) disc freq name(histasqs, replace)

graph combine histasqs asqage, col(1) fxsize(80) imargin (0 0 0 0) ///

name(supp_fig_A, replace) ///

subtitle("Supplementary figure A: ASQ score by" ///

"allocation group and age at follow-up") ///

note("ASQ: Ages and stages questionnaire") ///

iscale(*.8)

graph export supp_fig_A.tif, width(2000) replace

/// End of do-file

/* Do-file containing the analyses of premature for the manuscript "Non-specific

effects of BCG-vaccination on child psychomotor development: results from The

Danish Calmette Study - a randomised clinical trial"

edited by JK 25.11.15

*/

cd H:\forsk\csdevelopment

/////////////

//// Premature dataset 6, 12, 22 mo

////////////

use premature6modevelopment, clear

drop asqimp

rename asqage asq1age

rename asqs asq1s

rename coms coms1

rename fms fms1

rename gms gms1

rename pss pss1

rename psds psds1

merge 1:1 ridbarn using premature22modevelopment, nogen

drop asqimp

rename asqage asq3age

rename asqs asq3s

rename coms coms3

rename fms fms3

rename gms gms3

rename pss pss3

rename psds psds3

merge 1:1 ridbarn using bcgdevelopment, nogen

drop if premature == 2

drop asqimp

rename asqage asq2age

rename asqs asq2s

rename coms coms2

rename fms fms2

rename gms gms2

rename pss pss2

rename psds psds2

///////////

////// Results

//////////

// Responder, partial resp, non-resp:

gen asq1responder = .

replace asq1resp = 1 if asq1s != .

replace asq1resp = 0 if asq1s == .

gen asq1rbinary = 1 if asq1res == 1

replace asq1rbinary = 2 if asq1res !=1

lab var asq1rbin "binary asqresponder, 1= responder, 2 = partial and non-resp"

gen asq2responder = .

replace asq2resp = 1 if asq2s != .

replace asq2resp = 0 if asq2s == .

gen asq2rbinary = 1 if asq2res == 1

replace asq2rbinary = 2 if asq2res !=1

lab var asq2rbin "binary asqresponder, 1= responder, 2 = partial and non-resp"

/////

// Analyses

////

regress asq1s b2.rtreat asq1age i.BCG_freg02a

regress asq2s b2.rtreat asq2age i.BCG_freg02a

regress asq3s b2.rtreat asq3age i.BCG_freg02a

// Sensitivity analyses, adjusting for potential predictors of missingness:

* sex site moryob barnnr gadage BCG_inkl03 BCG_inkl05 BCG_inkl06 BCG_inkl15

merge m:1 rid using H:\forsk\csdata\acinkl, nogen keep(3)

regress asq1s b2.rtreat asq1age i.BCG_freg02a i.sex i.site moryob i.barnnr gadage i.BCG_inkl03 i.BCG_inkl05 i.BCG_inkl06 i.BCG_inkl15

regress asq2s b2.rtreat asq2age i.BCG_freg02a i.sex i.site moryob i.barnnr gadage i.BCG_inkl03 i.BCG_inkl05 i.BCG_inkl06 i.BCG_inkl15

regress asq3s b2.rtreat asq3age i.BCG_freg02a i.sex i.site moryob i.barnnr gadage i.BCG_inkl03 i.BCG_inkl05 i.BCG_inkl06 i.BCG_inkl15

////

misschk asq1s asq2s asq3s, gen(asqmiss)

snapshot save

merge m:1 rid using H:\forsk\csdata\acinkl, nogen keep(3)

keep ridbarn rtreat BCG_freg02a fold asqmissnum site sex morbcg asq1s asq1age ///

asq2s asq2age asq3s asq3age coms1 gms1 fms1 pss1 psds1 coms2 gms2 fms2 ///

pss2 psds2 coms3 gms3 fms3 pss3 psds3 moryob barnnr freg_sys_site ///

gadage BCG_inkl03 BCG_inkl05 BCG_inkl06 BCG_inkl07 BCG_inkl08 BCG_inkl10 ///

BCG_inkl15

rename asq1s asqs1

rename asq1age asqage1

rename asq2s asqs2

rename asq2age asqage2

rename asq3s asqs3

rename asq3age asqage3

reshape long asqage asqs asqimp coms gms fms pss psds, i(ridbarn BCG_freg02a fold asqmissnum ///

rtreat site sex moryob barnnr gadage ) j(followup)

lab var followup "Follow-up number"

snapshot save

/// Test for overall BCG effect at 6, 12, and 22 mo:

xtmixed asqs b2.rtreat i.followup asqa BCG_freg02a ||ridbarn:, noconstant residuals(unstructured, t(followup))

// Sensitivity: adjustering for predictors from MI:

regress asqs b2.rtreat#i.followup i.followup asqa i.BCG_freg02a i.sex i.site moryob ///

i.barnnr gadage i.BCG_inkl03 i.BCG_inkl05 i.BCG_inkl06 i.BCG_inkl15

/* 6.11.15: Figure 2 - premature sub-domains */

keep ridbarn rtreat coms - asqs asqa followup

rename coms v1

rename fms v2

rename gms v3

rename pss v4

rename psds v5

rename asqs v6

rename asqa v7

g n=_n

reshape long v, i(n) j(vnum)

label define vlab 1 "Coms" 2 "Fms" 3 "Gms" 4 "Pss" 5 "Psds" 6 "ASQ score" 7 "Age at followup"

label values vnum vlab

label define followuplab 1 "6 months" 2 "12 months" 3 "22 months"

label values followup followuplab

recode rtreat (1 = 2) (2 = 1), gen(treat)

label define treatlab 1 "Control" 2 "BCG"

label values treat treatlab

graph box v if vnum < 6 & followup == 1, over(treat) over(vnum) asy legend(label(2 "BCG") label(1 "Control")) ///

ytitle("Score") title("ASQ sub-domain 6 months") name(ASQ_subdomain_6, replace)

graph box v if vnum < 6 & followup == 2, over(treat) over(vnum) asy legend(label(2 "BCG") label(1 "Control")) ///

ytitle("Score") title("ASQ sub-domain 12 months") name(ASQ_subdomain_12, replace)

graph box v if vnum < 6 & followup == 3, over(treat) over(vnum) asy legend(label(2 "BCG") label(1 "Control")) ///

ytitle("Score") title("ASQ sub-domain 22 months") name(ASQ_subdomain_22, replace)

graph box v if vnum == 6, over(treat) over(vnum) over(followup) asy legend(label(2 "BCG") label(1 "Control")) ///

ytitle("Score") title("ASQ score at 6, 12 and 22 months") name(ASQ_scores, replace)

graph combine ASQ_subdomain_6 ASQ_subdomain_12 ASQ_subdomain_22 ASQ_scores, ///

subtitle("Figure 2: Box-plots of ASQ sub-domain scores and total" ///

"ASQ scores at 6, 12, and 22 months for premature children") ///

note("ASQ: Ages and stages questionnaire. Coms: Communication score. Fms: Fine motor score." ///

"Gms: Gross motor score. Pss: Problem solving score." ///

"Psds: Personal and social development score. BCG: Bacillus Calmette-Guérin vaccine.") ///

imargins (0 0 0 0) iscale(*.7) name(fig_2, replace)

graph export fig_2.tif, width(2000) replace

/// End of do-file
